# Gender differences in the mental health impact of the COVID-19 lockdown: Longitudinal evidence from the Netherlands

**DOI:** 10.1016/j.ssmph.2021.100878

**Published:** 2021-07-24

**Authors:** A. Vloo, R.J.M. Alessie, J.O. Mierau, Lifelines Corona Research Initiative, Marike H. Boezen, Jochen O. Mierau, Lude Franke, Jackie Dekens, Patrick Deelen, Pauline Lanting, Judith M. Vonk, Ilja Nolte, Anil P.S. Ori, Annique Claringbould, Floranne Boulogne, Marjolein X.L. Dijkema, Henry H. Wiersma, Robert Warmerdam, Soesma A. Jankipersadsing

**Affiliations:** a Department of Economics, Econometrics & Finance, Faculty of Economics & Business, University of Groningen, Groningen, the Netherlands; b Aletta Jacobs School of Public Health, Groningen, the Netherlands; c Department of Genetics, University of Groningen, University Medical Center Groningen, Groningen, the Netherlands; d Department of Epidemiology, University of Groningen, University Medical Center Groningen, Groningen, the Netherlands; e Department of Psychiatry, University of Groningen, University Medical Center Groningen, Groningen, the Netherlands; f Center of Development and Innovation, University of Groningen, University Medical Center Groningen, Groningen, the Netherlands; a Department of Economics, Econometrics & Finance, Faculty of Economics & Business, University of Groningen, Groningen, the Netherlands; b Aletta Jacobs School of Public Health, Groningen, the Netherlands

**Keywords:** COVID-19, Pandemic, Lockdown, Mental health, Depression, Anxiety, Gender differences

## Abstract

Recent contributions highlighted gender differences in the mental health consequences of COVID-19 lockdowns. However, their cross-sectional designs cannot differentiate between pre-existing gender differences and differences induced by lockdowns. Estimating fixed-effects models using longitudinal data from the Lifelines biobank and cohort study with repeated mental health measurements throughout the lockdown, we overcome this caveat. Significant gender differences in mental health during the lockdown were found, where women experienced more depression symptoms and disorders and men experienced more anxiety symptoms and disorders stemming from the lockdown. Policymakers need to keep in mind that the COVID-19 lockdowns have different effects on mental health for men and women.

## Highlights

• Mental health consequences of the COVID-19 pandemic appear to show strong gender differences.
• Current studies using cross-sectional data cannot differentiate between pre-existing and pandemic induced gender differences.
• Using longitudinal data we show that the pandemic had different mental health impacts on men and women.
• Women experienced more depression, while men experienced more anxiety.

## 1 Introduction

Although lockdowns are necessary to limit the occurrence of new COVID-19 cases and deaths, prolonged home confinement impacts both physical and mental health (Wang, Pan, et al., 2020; WHO, 2020). Potential results of quarantine include depressive symptoms, anxiety, insomnia and acute stress disorders (Brooks et al., 2020). Holmes et al. (2020) state: “It is already evident that the direct and indirect psychological and social effects of the coronavirus disease 2019 (COVID-19) pandemic are pervasive and could affect mental health now and in the future.” (p. 547). In line with this, a longitudinal study of the United States shows a substantial increase in depression and anxiety levels in the US during the emergence of the lockdown restrictions (Daly & Robinson, 2021), and a study from Holman et al. (2020) shows that depressive symptoms significantly increased over time between March and April 2020 in the US.

Existing literature during the COVID-19 pandemic reports that women, young people and people with low socioeconomic status (SES) report more problems regarding mental health issues as depression and anxiety (Ahmed et al., 2020; Gualano et al., 2020). This, however, is also true without the presence of the lockdown (Gao et al., 2020; Kiely et al., 2019; Ritchie & Roser, 2018), and is, therefore, not necessarily an effect of the lockdown on mental health. Therefore, we study whether the COVID-19 lockdown induces additional mental health differences between men and women. Investigating how the gender differences in mental health evolve during the COVID-19 pandemic, will help us understand the consequences of the lockdown to structural gender differences.

Using data from the Lifelines biobank and cohort study, containing data before, during and after the first COVID-19 lockdown for individuals aged 18 or older, we investigate the effects of the COVID-19 lockdown on gender differences in mental health of people living in Northern Netherlands. As mentioned, improving upon existing literature, we have access to longitudinal data, which enables us to draw causal interpretations of the lockdown on mental health differences in gender, which is not possible using cross-sectional data, which considers only one point in time (this has been done frequently as will be discussed in Section 2). For mental health we consider four measures: the number of depression symptoms, the number of anxiety symptoms, the occurrence of major depressive disorders and the occurrence of generalized anxiety disorders. In addition, we inspect how age, SES, marital status and children are related to gender and mental health, since evidence suggests that age and SES are important factors of risk during the COVID-19 pandemic (Ji Kang & In Jung, 2020; Khazanchi et al., 2020), and marital status and children are related to the state of mental health (Gualano et al., 2020).

In the next section, we review the existing literature concerning mental health in the COVID-19 pandemic. In Section 3 we describe the data we used and address potential selection bias problems. Section 4 shows the empirical analysis, from which the corresponding results can be found in Section 5. Finally, in Section 6 we discuss limitations of this study and in Section 7 we provide the conclusions.

## 2 Literature review

Most of the current research studying mental health consequences of COVID-19 lockdown measures is based on cross-sectional data settings. We summarize the main results for various countries in Table 1. These results are mainly from researchers in the United States, China and Italy, but we also have results from the United Kingdom, Spain, and Austria. From Table 1 we observe the Unites States reported that between 10.1% and 50.3% suffers from depression issues and between 7.1% and 45.4% suffers from anxiety issues. Italy reported that between 24.2% and 32.4% suffered from depression issues, and between 18.7% and 32.6% suffered from anxiety issues. Similarly, China reports that between 16.5% and 37.1% suffers from depression issues and between 12.9% and 35.1% from anxiety issues. Spain and Austria report 18.7% and 21.0% suffering from depression symptoms, and 21.6% and 19.0% suffering from anxiety symptoms, respectively, whereas the United Kingdom reports that only 7.6% of the sample suffers from depression issues and 10.2% suffers from anxiety issues during the COVID-19 pandemic. Two studies investigating multiple countries find 16.1% and 23.9% suffering from depression issues, and 22.2% and 27.8% suffering from anxiety issues. Most of these researchers found significant gender differences for both depression and anxiety issues, where women experience significantly more issues than men. Furthermore, all researchers who checked for significant age differences found these, where young subjects indicate to have more depression and anxiety issues than older subjects.

**Table 1:** Summary of results of various papers.

Country: Paper	Depression			Anxiety		
	Frequency (%)	Gender differences	Age differences	Frequency (%)	Gender differences	Age differences
US:						
Meyer et al., 2020	10.1	–	–	7.1	–	–
Wang et al., 2020	48.1	Yes	–	38.5	Yes	–
Liu et al., 2020	43.3	No	–	45.4	No	–
Rudenstine et al., 2021	50.3	Yes	Yes	41.3	Yes	Yes
Italy:						
Gualano et al., 2020	24.7	No	Yes	23.2	Yes	Yes
Cellini et al., 2020	24.2	–	–	32.6	–	–
Mazza et al., 2020	32.4	Yes	–	18.7	Yes	Yes
China:						
Ahmed et al., 2020	37.1	No	Yes	29.0	No	Yes
Huang and Zhao, 2020	20.1	No	Yes	35.1	No	Yes
Lei et al., 2020	22.4	Yes	Yes	12.9	Yes	Yes
Wang et al., 2020	16.5	Yes	–	28.8	Yes	–
UK:						
White and Van der Boor, 2020	7.6	No	–	10.2	Yes	–
Spain:						
González-Sanguino et al., 2020	18.7	Yes	Yes	21.6	Yes	Yes
Austria:						
Pieh et al., 2020	21.0	Yes	Yes	19.0	Yes	Yes
Multiple countries:						
Pouso et al., 2021	23.9*	Yes	Yes	27.8*	Yes	Yes
Barzilay et al., 2020	16.1	Yes	–	22.2	Yes	–

∗weighted averages.

As for SES, due to the COVID-19 pandemic, unemployment is increasing and people in a low-income category are hardest hit by the pandemic (Burström & Tao, 2020). Research in Austria also shows that people without work and people with low income are more prone to mental health issues (Pieh et al., 2020). It is also worth noticing that Gualano et al. (2020) showed that marital status and children are related to the state of mental health.

More studies like the ones mentioned can be found for other countries (Ahmad et al., 2020; Elmer et al., 2020; Moghanibashi-Mansourieh, 2020; Moreira et al., 2020; Rossi et al., 2020; Ueda et al., 2020; Zhang et al., 2020), but most researchers agree: Women, young people and people with low SES report more problems regarding mental health issues as depression and anxiety under COVID-19 restrictions.

The main limitation of these studies is the cross-sectional design, which restricts causal interpretations. As a result, we cannot state that the differences between genders and certain (socio-economic) subgroups are solely due to lockdown. Differences in mental health may exist even when the lockdown is not present. In fact, several researchers show that gender, age and SES are generally good predictors for a subject's mental health outcome (Angelini et al., 2018; Callander, 2016; De Graaf et al., 2012; Gao et al., 2020; Kiely et al., 2019; Ritchie & Roser, 2018; Silbersdorff & Schneider, 2019). For example, Kiely et al. (2019) provided evidence that women generally have significant worse mental health when assessed in terms of depression and anxiety. In order to overcome the caveats of existing literature, we use panel data to distinguish between gender effects stemming from the COVID-19 lockdown and gender differences in mental health outcomes in general.

## 3 Data

Lifelines is a multi-disciplinary prospective population-based cohort study examining in a unique three-generation design the health and health-related behaviors of 167,729 persons living in the Northern Netherlands. It employs a broad range of investigative procedures in assessing the biomedical, sociodemographic, behavioral, physical and psychological factors which contribute to the health and disease of the general population, with a special focus on multi-morbidity and complex genetics.

The first lockdown in the Netherlands started on March 16, 2020, when places including, but not limited to, schools, sporting facilities, restaurants and cafe's were closed. The first COVID-19 lockdown ended on June 1, 2020, when facilities were allowed to reopen under strict conditions. On March 30, 2020, adult Lifelines participants were invited to participate in the first round of the COVID-19 questionnaire, after this, questionnaires were sent out (bi-)weekly. For more details about the Lifelines COVID-19 cohort, we refer to McIntyre et al. (2021).

In this paper, data of the first eleven COVID-19 questionnaires were used. Furthermore, we used the last available wave of data before COVID-19 as starting point. Data in this wave is gathered in years 2014–2017, i.e. between three and six years before the COVID-19 questionnaires.

The main variables of interest, i.e. the measures of mental health, are the number of depression and anxiety symptoms and the occurrence of major depressive and generalized anxiety disorders. These symptoms are asked by means of the Mini International Neuropsychiatric Interview (MINI) in the COVID-19 questionnaires. We use the DSM-5 specification to determine depression and anxiety based on certain symptoms (Kernberg, 2013).

Another variable of interest is gender, but we are also interested in age, SES, marital status and the number of children at home, which we will use in combination with gender to discover differences between men and women for different categories of these variables.

We calculated current age as 2020 (current year) minus the year of birth of the individual. Age categories are: 18–30 years old, 31–50 years old, 51–65 years old, and 66 years old and older. Note that by this calculation, current age is a time invariant variable, i.e. current age is the same throughout the first COVID-19 lockdown.

For SES we used two different measures: the highest level of education achieved and net income per month. The categories for education are based on the last assessment before the COVID-19 pandemic started, since no such information is available during the COVID-19 pandemic. However, income is categorized according to the mean of two moments during the COVID-19 lockdown at which net income per month was available, time period 8 and 10. For highest education achieved we distinguish four categories based on the highest level of education using the International Standard Classification of Education (ISCED) (UNESCO Institute for Statistics, 2012): none or primary education (ISCED 0 or 1); lower secondary vocational or junior general secondary education (ISCED 2); secondary vocational or senior general secondary education (ISCED 3); and higher vocational or university education (ISCED 6, 7 or 8). For net income per month we distinguish five categories: less than €1000,-; between €1000,- and €2000,-; between €2000,- and €3000,-; between €3000,- and €4000,-; and more than €4000,-. Note again that highest education achieved and net income per month are time invariant variables.

Marital status is also a time invariant variable since it was only asked before the COVID-19 lockdown. Marital status categories are: married; registered partnership; in relationship, but living apart; single; and other. For the number of children at home we distinguish between children aged between zero and 12 years old, and between 13 and 18 years old. These variables are time varying since these were asked various times during the COVID-19 pandemic and are, therefore, not necessarily constant during the lockdown. We have categories: zero children; one or two children; three or four children; and five or more children.

### 3.1 Summary characteristics

We calculated summary statistics based on our sample as a whole, the sample of respondents and the sample of non-respondents of the COVID-19 questionnaires. Non-respondents are individuals who were invited to participate in the COVID-19 questionnaires but did not respond to any of those questionnaires. The characteristics can be found in Table 2. These summary statistics are from the last available wave before the COVID-19 questionnaires in order to compare respondents and non-respondents of the COVID-19 questionnaires. From the last column, i.e. characteristics from the sample as a whole, we observe that in total 134,434 individuals were invited to participate in the COVID-19 questionnaires. Almost 3% reported to have major depressive disorders and over 5% reported to have generalized anxiety disorders. The mean number of depression and anxiety symptoms are 0.27 and 0.32 before the COVID-19 outbreak, respectively. Comparing those values between respondents and non-respondents of the COVID-19 questionnaires, we see that respondents have significantly less depression and anxiety symptoms and disorders than non-respondents and that respondents rate their health better. This indicates that we face a selection problem, which we will discuss in more detail in section 3.2.

**Table 2:** Characteristics of respondents and non-respondents to the COVID-19 questionnaires.

Variable	Respondents	Non-respondents	P-value	Total
Number of observations, (%)	72,098 (53.63)	62,336 (46.37)		134,434

Number of depression symptoms before COVID-19, mean (sd)	0.25 (1.11)	0.32 (1.26)	*P* < 0.0001	0.27 (1.17)
Major depressive disorder before COVID-19, %	2.54	3.38	*P* < 0.0001	2.83
Number of anxiety symptoms before COVID-19, mean (sd)	0.30 (1.24)	0.36 (1.37)	*P* < 0.0001	0.32 (1.28)
Generalized anxiety disorder before COVID-19, %	4.95	5.98	*P* < 0.0001	5.31
Self-rated health (1 = excellent, … , 5 = bad), mean (sd)	2.63 (0.80)	2.70 (0.80)	*P* < 0.0001	2.66 (0.80)

Men, %	39.31	43.99	*P* < 0.0001	41.48
Current age, mean (sd)	56.57 (12.19)	50.76 (12.27)	*P* < 0.0001	55.22 (12.50)
Marital status at last visit, %				
Married	66.53	61.89	*P* < 0.0001	64.87
Registered partner- ship	16.54	20.24	*P* < 0.0001	17.86
In relationship but living apart	4.94	5.60	*P* < 0.0001	5.18
Single	11.09	11.30	*P* = 0.3409	11.16
Other	0.90	0.97	*P* = 0.3697	0.93
BMI at last visit, mean (sd)	26.08 (4.25)	26.11 (4.26)	*P* = 0.4750	26.09 (4.25)
Smoking at last visit, %				
Never	46.52	47.06	*P* = 0.1686	46.68

Ex	40.02	34.18	*P* < 0.0001	38.30
Current	13.46	18.75	*P* < 0.0001	15.06
Number of people in household, mean (sd)	2.76 (1.23)	3.03 (1.30)	*P* < 0.0001	2.85 (1.26)

Highest education at last visit, %				
None	0.29	0.65	*P* < 0.0001	0.42
Primary	0.92	1.62	*P* < 0.0001	1.17
Lower or preparatory secondary vocational	10.84	13.74	*P* < 0.0001	11.88
Junior general secondary	14.13	13.54	*P* = 0.0180	13.92
Secondary vocational or work-based learning pathway	28.53	31.44	*P* < 0.0001	29.57
Senior general secondary education or pre-university secondary	8.12	7.08	*P* < 0.0001	7.75
Higher vocational	28.12	24.21	*P* < 0.0001	26.72
University	7.44	6.22	*P* < 0.0001	7.00
Other	1.62	1.49	*P* = 0.1397	1.57
Net income per month at last visit (1 = Less than €750, … , 11 = More than €5000), mean (sd)	5.23 (1.76)	4.98 (1.85)	*P* = 0.1340	5.23 (1.76)
Work situation at last visit (before COVID-19), %				
student	0.48	0.64	*P* < 0.0001	0.54
paid work	72.03	77.27	*P* < 0.0001	73.91
retired	13.38	8.07	*P* < 0.0001	11.48
unfit for work	2.92	3.06	*P* = 0.7771	2.97

unemployed/look- ing for work	3.54	3.37	*P* < 0.0001	3.48
other	7.64	7.60	*P* = 0.0069	7.63

The mean number of depression and anxiety symptoms and disorders are represented graphically in Fig. 1a and Fig. 1b, respectively. In these figures, and in the next figures, we use only the individuals who answered at least one of the COVID-19 questionnaires conditional on having answered the last available questionnaire before the COVID-19 pandemic. Time point zero indicates the last wave before the lockdown started. It is worth noticing that this is gathered in years 2014–2017, i.e. between three and six years before the COVID-19 questionnaires.

**Fig. 1a:**
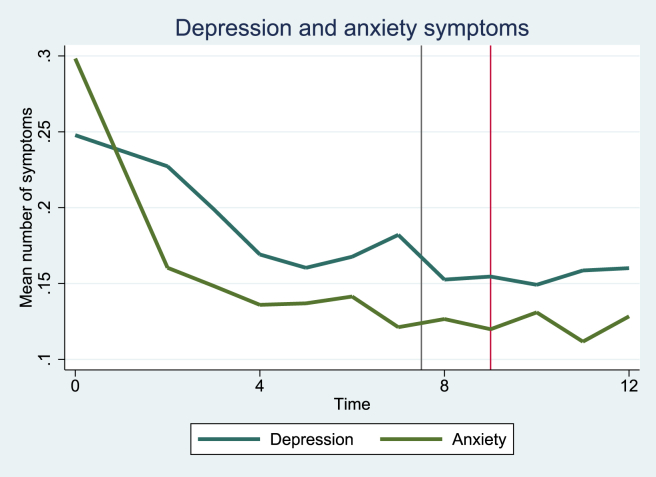
Depression and anxiety symptoms. Note: the vertical gray line indicates the reopening of elementary schools (May 11, 2020); the vertical red line indicates the end of the lockdown (June 1, 2020). (For interpretation of the references to colour in this figure legend, the reader is referred to the Web version of this article.)

**Fig. 1b:**
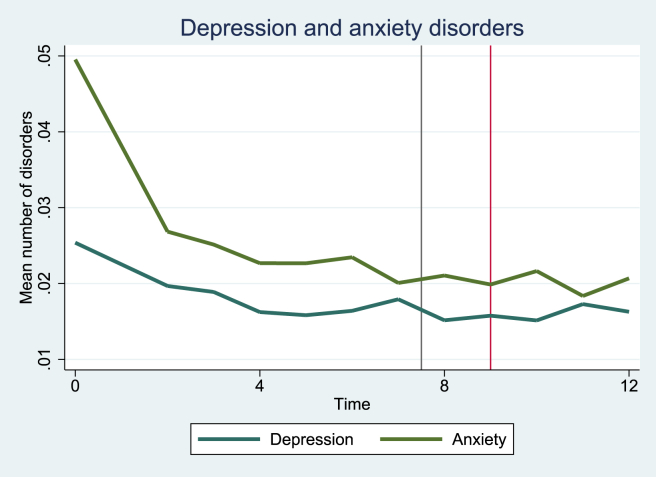
Major depressive and generalized anxiety disorders. Note: the vertical gray line indicates the reopening of elementary schools (May 11, 2020); the vertical red line indicates the end of the lockdown (June 1, 2020). (For interpretation of the references to colour in this figure legend, the reader is referred to the Web version of this article.)

After time point zero, each COVID-19 questionnaire is considered a different point in time starting at time two. In these figures, as well as in the next figures, the vertical red line indicates the end of the lockdown, i.e. June 1, 2020, and the vertical gray line indicates when elementary schools reopened, i.e. May 11, 2020.

Surprisingly, we observe that both the mean number of depression and anxiety symptoms and disorders decrease when the lockdown starts and never return to their initial high state before the lockdown. As mentioned above, time point zero is measured at least three years before the outbreak of COVID-19. In these years a lot might have happened that explains this sudden drop of depression and anxiety symptoms and disorders. However, sensitivity analyses excluding the initial pre-lockdown period leave our main results below unchanged (available on request).

From Fig. 1a and Fig. 1b it is also noteworthy that the decrease in anxiety symptoms and disorders is steeper than the decrease in depression symptoms and disorders at the start. Furthermore, the duration of the lockdown does not seem to matter to the state of mental health, i.e. over time, mental health becomes almost constant. 1

### 3.2 Gender

Let us now look into gender differences regarding mental health. Fig. 2a and Fig. 2b show the mean number of depression and anxiety symptoms by gender, respectively. We clearly observe a difference between men and women: women indicate more depression and anxiety symptoms than men. This is in line with results from the literature review. Contrasting to existing literature, we can now investigate trends. Without any formal statistical analysis we observe that the difference between men and women increases during the lockdown for depression but decreases during the lockdown for anxiety. Hence, women and men respond differently to the COVID-19 lockdown in terms of the number of depression and anxiety symptoms. Note how the mean number of depression and anxiety symptoms decreases over time for both men and women, and that the decrease at the start is steeper for men regarding depression and steeper for women regarding anxiety. 2

**Fig. 2a:**
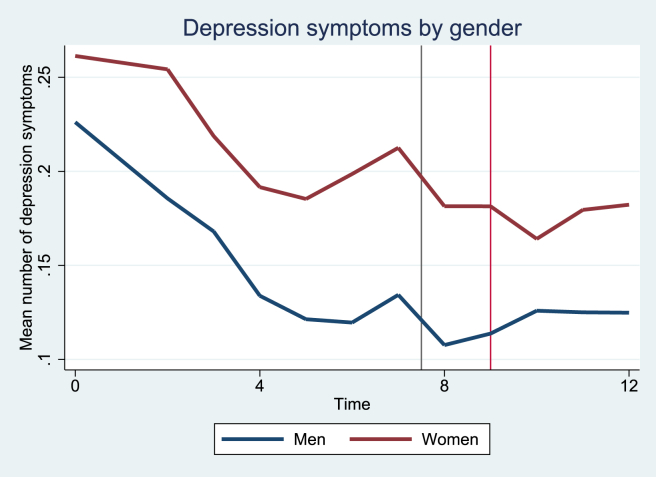
Depression symptoms by gender. Note: the vertical gray line indicates the reopening of elementary schools (May 11, 2020); the vertical red line indicates the end of the lockdown (June 1, 2020). (For interpretation of the references to colour in this figure legend, the reader is referred to the Web version of this article.)

**Fig. 2b:**
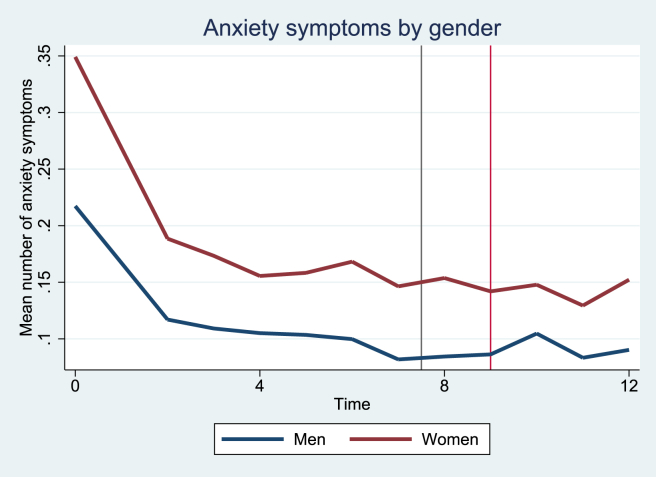
Anxiety symptoms by gender. Note: the vertical gray line indicates the reopening of elementary schools (May 11, 2020); the vertical red line indicates the end of the lockdown (June 1, 2020). (For interpretation of the references to colour in this figure legend, the reader is referred to the Web version of this article.)

**Fig. 2c:**
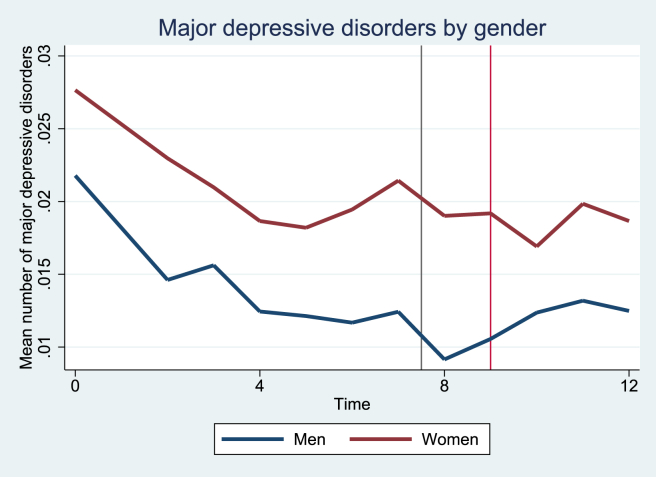
Major depressive disorders by gender. Note: the vertical gray line indicates the reopening of elementary schools (May 11, 2020); the vertical red line indicates the end of the lockdown (June 1, 2020). (For interpretation of the references to colour in this figure legend, the reader is referred to the Web version of this article.)

**Fig. 2d:**
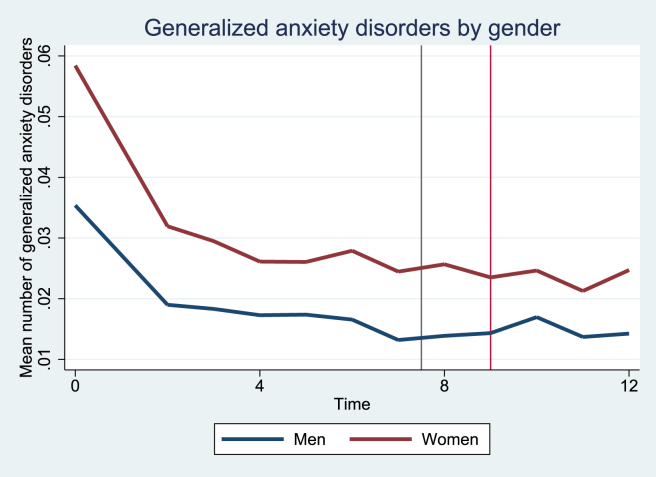
Generalized anxiety disorders by gender. Note: the vertical gray line indicates the reopening of elementary schools (May 11, 2020); the vertical red line indicates the end of the lockdown (June 1, 2020). (For interpretation of the references to colour in this figure legend, the reader is referred to the Web version of this article.)

Although this graphical analysis provides compelling evidence that clear gender differences exist regarding the trend of mental health; some problems arise regarding the interpretation of this graphical analysis. In this analysis we do not account for potential bias. We know sample selection bias exists since the mental health measures are significantly different for respondents and non-respondents as discussed before and as can be seen in Table 2: respondents of the COVID-19 questionnaires have significantly less depression and anxiety symptoms and disorders. We check if sample selection bias is still present when controlling for other characteristics. These other characteristics, which we also include in the model in our analysis, control some of the variation in depression and anxiety symptoms and disorders. Including these characteristics and a dummy variable indicating whether the individual responded to at least one of the COVID-19 questionnaires, in a model on our mental health measures, will show if being a respondent is still significant for mental health when other characteristics are taken into account. Fortunately, we observe that it is not, i.e. being a respondent of the COVID-19 questionnaires does not significantly impact the outcome variables measuring mental health when accounting for other characteristics of the individuals. Another selection bias we might face is attrition bias. Attrition bias occurs when respondents leave in a non-random way during the study, in contrast to sample selection bias which occurs before the study starts. We check for attrition bias by means of a method by Kapteyn et al. (2005) where we examine if respondents leave during the study in a non-random way. From the analysis we conclude that we do not suffer from attrition bias. Details on how we tested sample selection bias and attrition bias, and potential consequences if we do not correct for these selection biases can be found in Appendix 1.

## 4 Methodology

In order to further explore the graphical analysis, which showed that women seem to experience negative effects on depression from prolonged lockdown whereas the opposite is true for anxiety, we need to define appropriate models. The dependent variables of our models are the number of depression symptoms, the number of anxiety symptoms, the occurrence of major depressive disorders, and the occurrence of generalized anxiety disorders. For both the continuous and binary measures, we start by using a linear fixed effects model. 3 Note that time invariant variables such as gender, age category, education category and income category, will be omitted in the fixed effects model. However, we are interested in gender differences induced by the lockdown, for which we use gender interacted with the time period; this variable is time variant.

### 4.1 Base model

Let us consider the base model. We define for *i* = 1, *…*, *N* and *t* = 1, *…*, 12:

(1) $$y_{it}=x_{it}^{\prime }\beta +c_{i}+u_{it},$$

where *i* denotes individual subjects, *t* denotes the time period, *c*_*i*_ is the individual specific fixed effect, which captures time invariant regressors and is allowed to be correlated with ***x***_*it*_, and *u*_*it*_ is the idiosyncratic error. The standard errors are clustered at the individual level. The vector ***x***_*it*_ contains variables including time dummies and variables on time periods interacted with gender, age categories, education categories and income. We are mostly interested in the coefficients for time interacted with gender, from which we observe the additional gender differences in mental health due to the COVID-19 lockdown. After estimating the base model, we expand the base model by adding three-way interactions with gender, time and age; gender, time and SES; gender, time and marital status; and gender, time and number of children at home.

It is worth noticing that *t* = 1 corresponds to the time period before the lockdown; *t* = 2 until *t* = 9 correspond to the time period during the first COVID-19 lockdown; and *t* = 10 until *t* = 12 correspond to the time period after the first lockdown ended. Note that *t* = 1 will be omitted to avoid multicollinearity.

## 5 Results

The results regarding the interaction terms between gender and time, as can be seen in Table 3 (or in Table 3 in Appendix 2 including all variable estimates), are represented graphically in Fig. 3a, Fig. 3b, Fig. 3c, Fig. 3d, Fig. 3e, Fig. 3f, Fig. 3g, Fig. 3h.^4^ First we analyze results for depression symptoms and disorders. Secondly, we analyze results for anxiety symptoms and disorders. Next, we research joint significance of the coefficients to underline the previous results. Lastly, we discuss results obtained when adding three-way interactions.

**Table 3:** Results base model: interaction term gender and time period.

	(1)	(2)	(3)	(4)
	Depr. symptoms	Depr. disorder	Anx. symptoms	Anx. disorder
**Woman×**				
Time 2	0.0439*	0.00395	−0.0317	−0.00528
	(0.0179)	(0.00257)	(0.0186)	(0.00333)
Time 3	0.00272	−0.00177	−0.0423*	−0.00819*
	(0.0172)	(0.00255)	(0.0181)	(0.00327)
Time 4	0.0233	0.000568	−0.0450*	−0.00820*
	(0.0171)	(0.00249)	(0.0180)	(0.00320)
Time 5	0.0355*	0.00168	−0.0465**	−0.00909**
	(0.0168)	(0.00244)	(0.0174)	(0.00312)
Time 6	0.0491**	0.00288	−0.0399*	−0.00751*
	(0.0167)	(0.00248)	(0.0180)	(0.00322)
Time 7	0.0329	0.00179	−0.0410*	−0.00753*
	(0.0170)	(0.00249)	(0.0179)	(0.00317)
Time 8	0.0381*	0.00403	−0.0320	−0.00584
	(0.0170)	(0.00246)	(0.0179)	(0.00319)
Time 9	0.0463**	0.00485	−0.0492**	−0.00896**
	(0.0170)	(0.00252)	(0.0180)	(0.00318)
Time 10	0.0165	−0.000141	−0.0572**	−0.00961**

	(0.0173)	(0.00256)	(0.0189)	(0.00336)
Time 11	0.0118	−0.0000953	−0.0665***	−0.0113***
	(0.0179)	(0.00264)	(0.0185)	(0.00328)
Time 12	0.0199	−0.000607	−0.0533**	−0.00912**
	(0.0175)	(0.00258)	(0.0184)	(0.00325)

Standard errors in parentheses, clustered at individual level.

*p < 0.05, **p < 0.01, ***p < 0.001.

**Fig. 3a:**
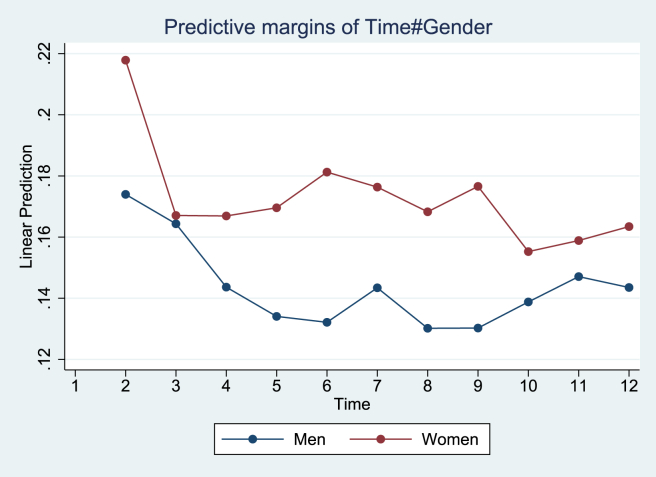
Average marginal effects of time on the number of depression symptoms by gender.

**Fig. 3b:**
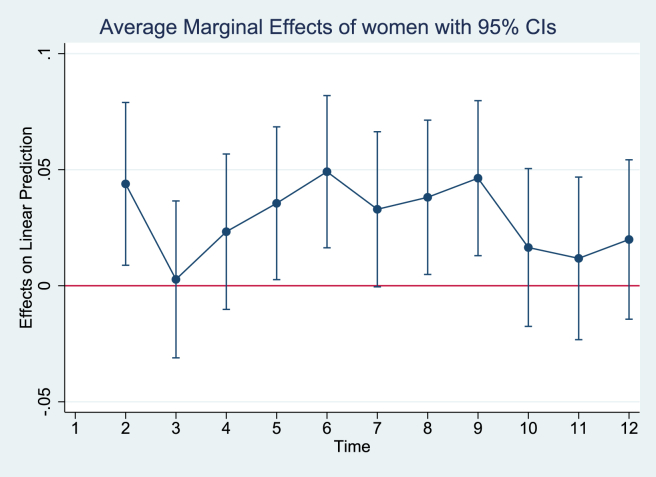
Difference in gender of average marginal effects of time on the number of depression symptoms. Note: the horizontal red line at zero is added since we are interested in effects different from zero. Positive effects indicate that women have more depression symptoms than men, vice versa for negative effects. (For interpretation of the references to colour in this figure legend, the reader is referred to the Web version of this article.)

**Fig. 3c:**
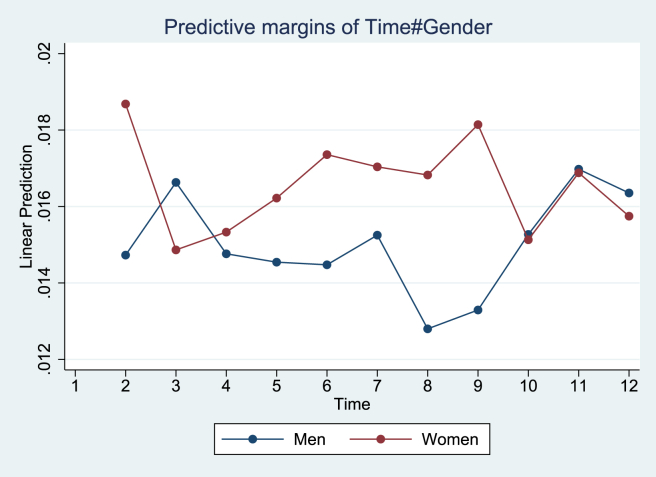
Average marginal effects of time on the occurrence of major depressive disorders by gender.

**Fig. 3d:**
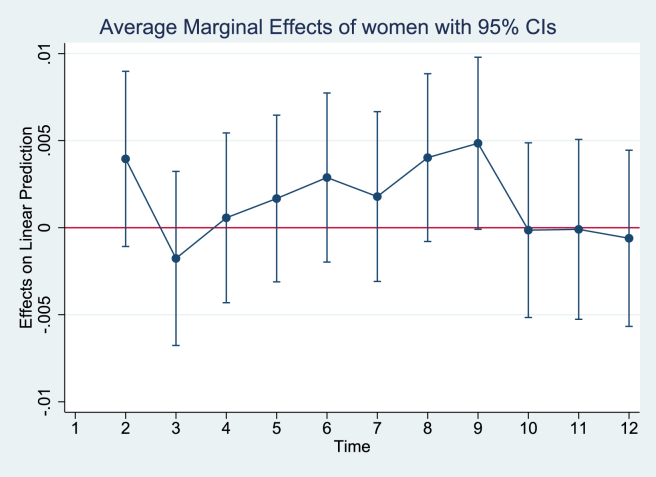
Difference in gender of average marginal effects of time on the occurrence of major depressive disorders. Note: the horizontal red line at zero is added since we are interested in effects different from zero. Positive effects indicate that women have more depression disorders than men, vice versa for negative effects. (For interpretation of the references to colour in this figure legend, the reader is referred to the Web version of this article.)

**Fig. 3e:**
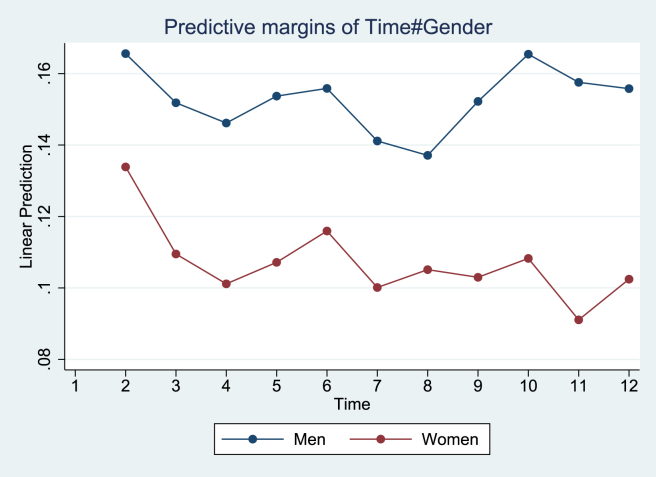
Average marginal effects of time on the number of anxiety symptoms by gender.

**Fig. 3f:**
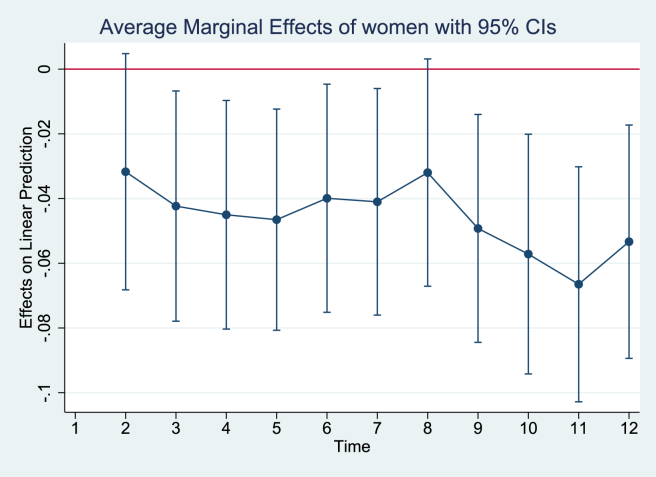
Difference in gender of average marginal effects of time on the number of anxiety symptoms. Note: the horizontal red line at zero is added since we are interested in effects different from zero. Positive effects indicate that women have more anxiety symptoms than men, vice versa for negative effects. (For interpretation of the references to colour in this figure legend, the reader is referred to the Web version of this article.)

**Fig. 3g:**
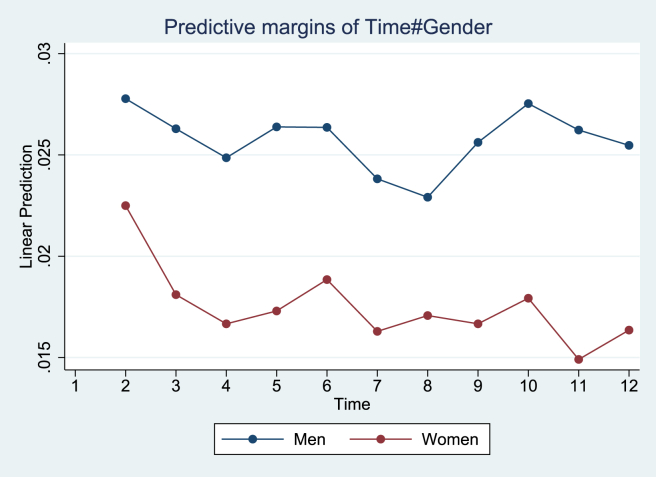
Average marginal effects of time on the occurrence of generalized anxiety disorders by gender.

**Fig. 3h:**
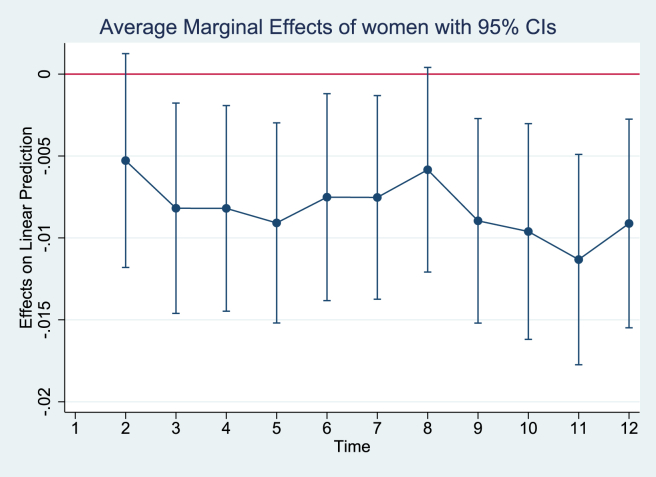
Difference in gender of average marginal effects of time on the occurrence of generalized anxiety disorders. Note: the horizontal red line at zero is added since we are interested in effects different from zero. Positive effects indicate that women have more anxiety disorders than men, vice versa for negative effects. (For interpretation of the references to colour in this figure legend, the reader is referred to the Web version of this article.)

### 5.1 Depression symptoms and disorders

From Fig. 3a it is clear that the effect of the time period on the number of depression symptoms differs by gender, as we also observed in Section 3. Let us now assess these gender differences in the number of depression symptoms. The results can be found in Fig. 3b, where the solid blue dots indicate the difference in the average marginal effects of time on the number of depression symptoms of women and men, and 95% confidence intervals are added to easily distinguish significant gender differences: when the confidence interval does not include the reference line at zero, the difference is statistically significant on a 5% level. Note that the differences per time period between men and women (the solid blue dots) have a value equal to the associated estimate in Table 3. Note again that the end of lockdown is at *t* = 9 and elementary schools reopened at *t* = 7.5. We observe in Fig. 3b that for some time periods, the confidence intervals do not include the reference line. Furthermore, the gender differences in the average marginal effects are all above the reference line at zero. Considering time period two, we can conclude that, compared to being in time period one, the effect of being in time period two on the number of depression symptoms is significantly larger for women than for men, ceteris paribus. In fact, as can be seen in Table 3, compared to being in time period one, the effect of being in time period two on the number of depression symptoms is for a women 4.4 percentage point (since the average marginal effect is 0.0439) higher than it is for men, ceteris paribus. Similarly, for time periods five, six, eight and nine, which all show a 95% confidence interval entirely above the reference line we can conclude that, compared to being in time period one, the effect of being in one of these time periods on the number of depression symptoms is significantly larger for women than for men, ceteris paribus. Therefore, women have significantly more depression symptoms stemming from the COVID-19 lockdown than men. Note that this widens the existing structural gender gap.

When analyzing results for the occurrence of major depressive disorders, we see again, in Fig. 3c, that the effect of the time period on the occurrence of major depressive disorders seems to differ by gender. However, when inspecting these gender differences in Fig. 3d we see that all 95% confidence intervals include the reference line, meaning that no significant gender differences regarding the occurrence of major depressive disorders on a 5% level exist, ceteris paribus.

We can conclude that, as opposed to men, women experience significantly more depression symptoms stemming from the COVID-19 lockdown, i.e. on top of pre-existing gender differences.

### 5.2 Anxiety symptoms and disorders

Both Fig. 3e and Fig. 3g show that the average marginal effects of the time period on the numbers of symptoms and on the occurrence of generalized anxiety disorders, differ by gender. Investigating these gender differences in Fig. 3f and Fig. 3h we see that the gender differences in average marginal effects are all below the reference line, and most 95% confidence intervals are also below this line for both the number of symptoms and the occurrence of disorders. This means that, compared to being in time period one, men experience signficantly more anxiety symptoms and generalized anxiety disorders in most periods after this than women, ceteris paribus. Therefore, men experience significantly more anxiety symptoms and disorders induced by the lockdown than women, on top of any pre-existing gender differences.

### 5.3 Joint significance

For each mental health measure, we also tested joint significance of the coefficients for gender interacted with time. We tested if the interactions are jointly significant in time periods two to nine (during the first COVID-19 lockdown), time periods 10 to 12 (after the lockdown), and time periods two to 12 (during and after the lockdown). When joint significance is found, we can state that men and women differ significantly in their reaction to the lockdown when interested in mental health. The results can be found in Table 4. First, considering the number of depression symptoms and the occurrence of major depressive disorders, we observe that the coefficients for gender interacted with time are jointly significant on a 5% level during the lockdown and during and after the lockdown. The joint significance of gender interacted with time on depression symptoms during the lockdown means that women respond differently, in our case worse, in terms of the number of depression symptoms than men during COVID-19 lockdown. Similarly for the period during and after the lockdown, and similarly for the occurrence of major depressive disorders.

**Table 4:** Joint significance of interaction between gender and time dummies (on 5% level).

	During	After	During and after
Depression symptoms	Yes (*p* = 0.0011)	No (*p* = 0.6694)	Yes (*p* = 0.0005)
Major depressive disorders	Yes (*p* = 0.0184)	No (*p* = 0.9895)	Yes (*p* = 0.0057)
Anxiety symptoms	No (*p* = 0.1991)	Yes (*p* = 0.0046)	No (*p* = 0.0534)
Generalized anxiety disorders	No (*p* = 0.1098)	Yes (*p* = 0.0073)	No (*p* = 0.0641)

Next considering the number of anxiety symptoms and the occurrence of generalized anxiety disorders, we observe that the coefficients of gender interacted with time are jointly significant for the number of anxiety symptoms and the occurrence of generalized anxiety disorders after the lockdown ended. Similar conclusions as above apply.

These results underline the previous results: Men and women differ significantly in their reaction to the COVID-19 lockdown when considering mental health, where women experience significantly more depression symptoms and disorders from the lockdown, whereas men experience significantly more anxiety symptoms and disorders from the lockdown.

### 5.4 Three-way gender interactions

As discussed in the methodology section, we expand the base model by adding three-way interactions between gender, time and age; gender, time and SES; gender, time and marital status; and gender, time and the number of children at home.

#### 5.4.1 Time, gender and age

Firstly, let us consider the interaction terms of gender, time and age in Fig. 4a, Fig. 4b, Fig. 4c, Fig. 4d. Whereas significant differences between gender for the effect of age on the number of depression symptoms or disorders are not found for any time period, Fig. 4d provides evidence that being man is related to more generalized anxiety disorders from time period 6 onwards in age category 18–30 years old, ceteris paribus. Hence, young men experience more anxiety disorders stemming from the COVID-19 lockdown than young women, which might be explained by the fact that young people and men tend to react worse to the lockdown than older people and women in general (Gebhard et al., 2020; Ji Kang & In Jung, 2020; Khazanchi et al., 2020).

**Fig. 4a:**
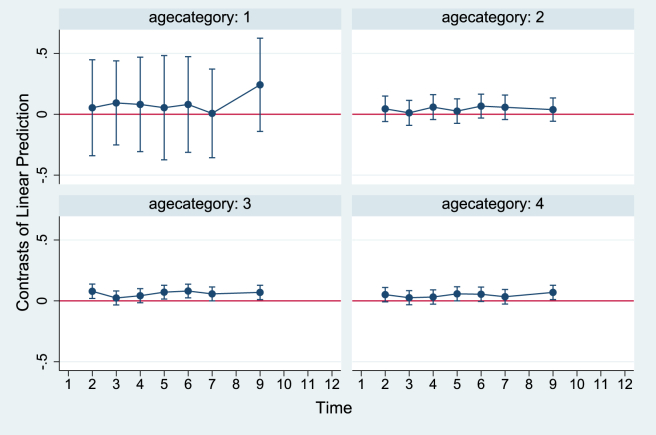
Difference in gender of average marginal effects of time on the number of depression symptoms by age. Note: the horizontal red line at zero is added since we are interested in effects different from zero. Positive effects indicate that women in a certain age category have more depression symptoms than men in the same age category, vice versa for negative effects. (For interpretation of the references to colour in this figure legend, the reader is referred to the Web version of this article.)

**Fig. 4b:**
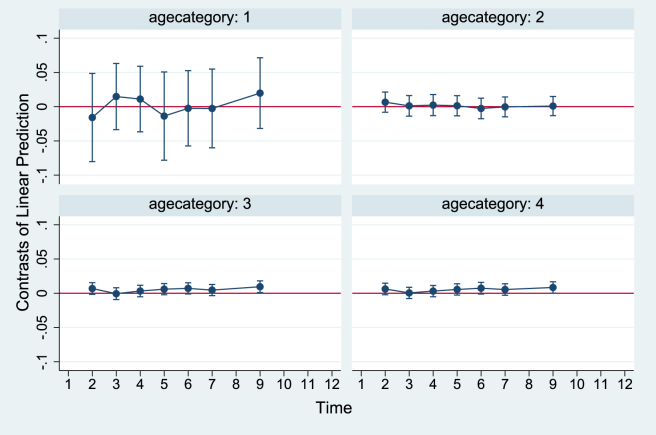
Difference in gender of average marginal effects of time on the occurrence of major depressive disorders by age. Note: the horizontal red line at zero is added since we are interested in effects different from zero. Positive effects indicate that women in a certain age category have more depression disorders than men in the same age category, vice versa for negative effects. (For interpretation of the references to colour in this figure legend, the reader is referred to the Web version of this article.)

**Fig. 4c:**
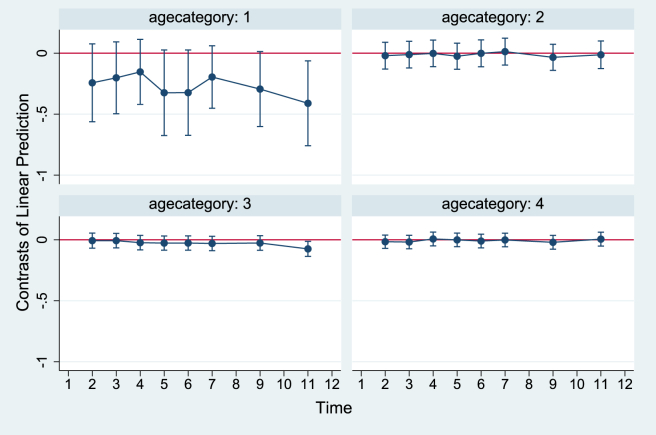
Difference in gender of average marginal effects of time on the number of anxiety symptoms by age. Note: the horizontal red line at zero is added since we are interested in effects different from zero. Positive effects indicate that women in a certain age category have more anxiety symptoms than men in the same age category, vice versa for negative effects. (For interpretation of the references to colour in this figure legend, the reader is referred to the Web version of this article.)

**Fig. 4d:**
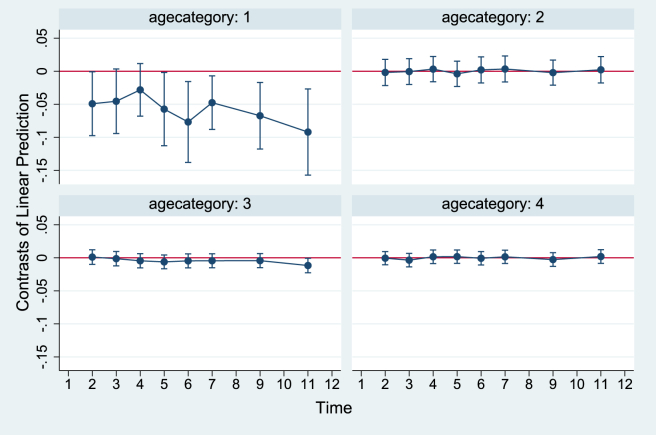
Difference in gender of average marginal effects of time on the occurrence of generalized anxiety disorders by age. Note: the horizontal red line at zero is added since we are interested in effects different from zero.Positive effects indicate that women in a certain age category have more anxiety disorders than men in the same age category, vice versa for negative effects. (For interpretation of the references to colour in this figure legend, the reader is referred to the Web version of this article.)

#### 5.4.2 Time, gender and education

Regarding the three-way interaction between gender, time and education, we observe in Fig. 5a, Fig. 5b, Fig. 5c, Fig. 5d that, whereas no significant differences exist between men and women for the effect of education on anxiety symptoms or disorders for any time period, we found evidence showing women with higher achieved education (especially higher vocational or university education) experience more depression symptoms and disorders than men with this education in almost all periods during and after the first COVID-19 lockdown, ceteris paribus. Hence, highly educated women experience more depression symptoms and disorders from the COVID-19 lockdown than highly educated men. A potential explanation might be that highly educated women experience more pressure to find and keep a job than lower educated women (Bussemakers et al., 2017; Samarakoon & Parinduri, 2015), whereas, on top of the fact that the COVID-19 pandemic limits job opportunities, the odds of finding and keeping a job are smaller for women than for men in general (Batz-Barbarich et al., 2018; McGinn & Oh, 2017; Petrongolo, 2019). This situation, i.e. wanting to find and keep a job with the knowledge that women are less likely to do this than men, might cause more mental health problems.

**Fig. 5a:**
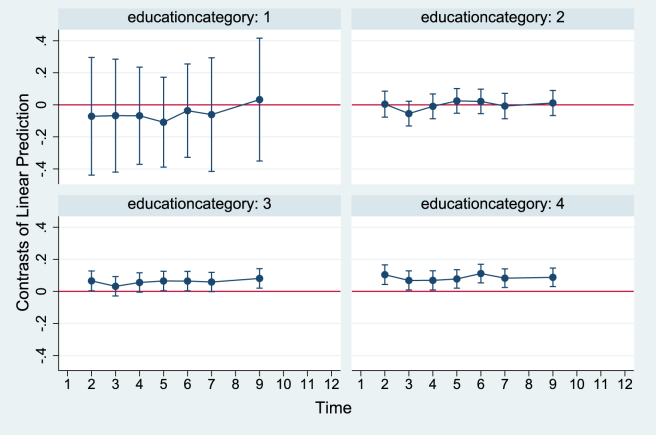
Difference in gender of average marginal effects of time on the number of depression symptoms by education. Note: the horizontal red line at zero is added since we are interested in effects different from zero. Positive effects indicate that women in a certain education category have more depression symptoms than men in the same education category, vice versa for negative effects. (For interpretation of the references to colour in this figure legend, the reader is referred to the Web version of this article.)

**Fig. 5b:**
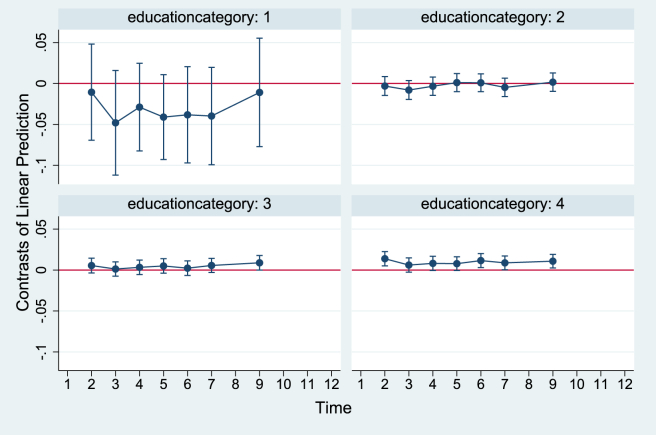
Difference in gender of average marginal effects of time on the occurrence of major depressive disorders by education. Note: the horizontal red line at zero is added since we are interested in effects different from zero. Positive effects indicate that women in a certain education category have more depression disorders than men in the same education category, vice versa for negative effects. (For interpretation of the references to colour in this figure legend, the reader is referred to the Web version of this article.)

**Fig. 5c:**
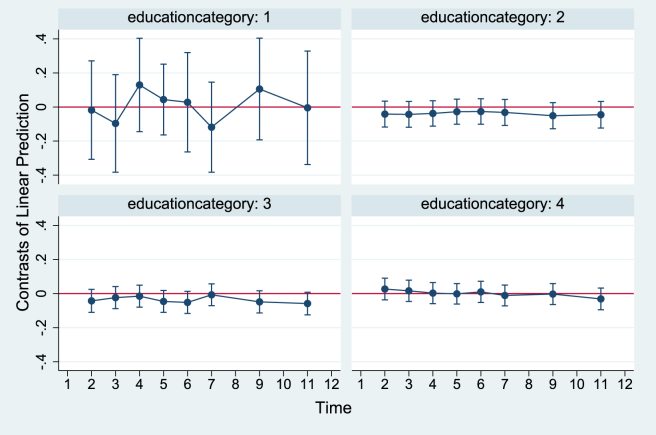
Difference in gender of average marginal effects of time on the number of anxiety symptoms by education. Note: the horizontal red line at zero is added since we are interested in effects different from zero. Positive effects indicate that women in a certain education category have more anxiety symptoms than men in the same education category, vice versa for negative effects. (For interpretation of the references to colour in this figure legend, the reader is referred to the Web version of this article.)

**Fig. 5d:**
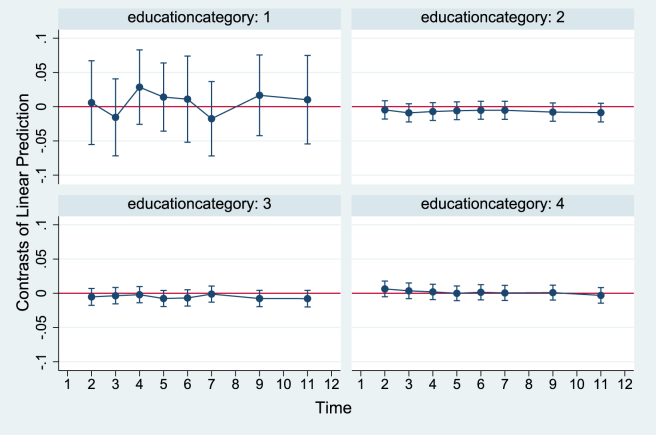
Difference in gender of average marginal effects of time on the occurrence of generalized anxiety disorders by education. Note: the horizontal red line at zero is added since we are interested in effects different from zero. Positive effects indicate that women in a certain education category have more anxiety disorders than men in the same education category, vice versa for negative effects. (For interpretation of the references to colour in this figure legend, the reader is referred to the Web version of this article.)

#### 5.4.3 Time, gender and income

Inspecting gender interacted with time and income, we observe from Fig. 6a, Fig. 6b, Fig. 6c, Fig. 6d that women in low income categories have significantly more depression symptoms and disorders than men in a low income category when in lockdown, ceteris paribus. For anxiety symptoms and disorders we find that women in the middle income category have significantly less anxiety symptoms and disorders than men in this category in all periods, ceteris paribus, i.e. prolonged lockdown is worse in terms of anxiety for men in the middle income class than for women in this class.

**Fig. 6a:**
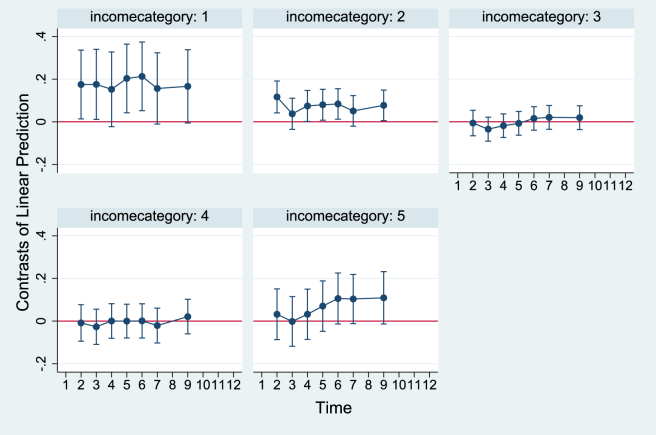
Difference in gender of average marginal effects of time on the number of depression symptoms by income. Note: the horizontal red line at zero is added since we are interested in effects different from zero. Positive effects indicate that women in a certain income category have more depression symptoms than men in the same income category, vice versa for negative effects. (For interpretation of the references to colour in this figure legend, the reader is referred to the Web version of this article.)

**Fig. 6b:**
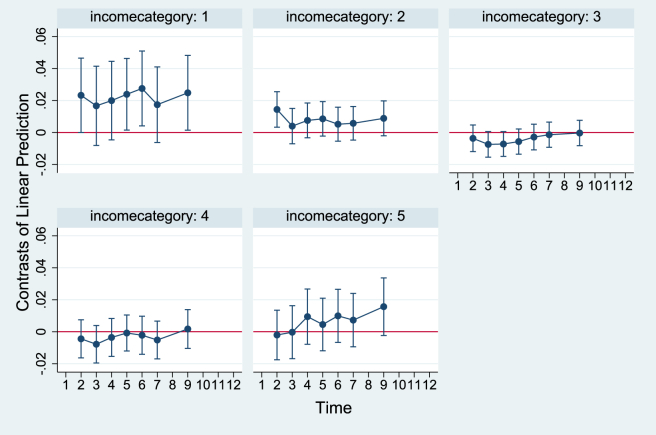
Difference in gender of average marginal effects of time on the occurrence of major depressive disorders by income. Note: the horizontal red line at zero is added since we are interested in effects different from zero. Positive effects indicate that women in a certain income category have more depression disorders than men in the same income category, vice versa for negative effects. (For interpretation of the references to colour in this figure legend, the reader is referred to the Web version of this article.)

**Fig. 6c:**
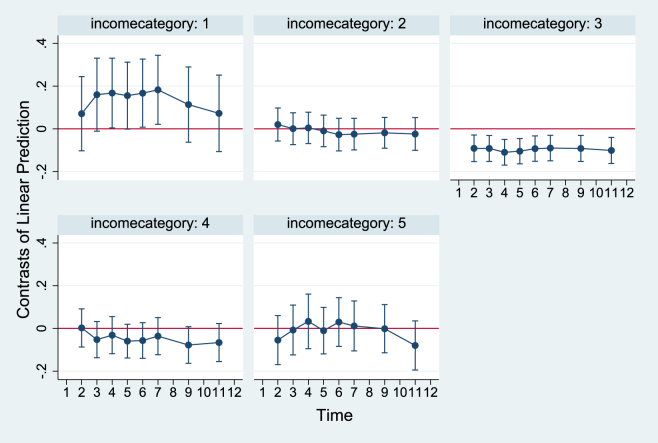
Difference in gender of average marginal effects of time on the number of anxiety symptoms by income. Note: the horizontal red line at zero is added since we are interested in effects different from zero. Positive effects indicate that women in a certain income category have more anxiety symptoms than men in the same income category, vice versa for negative effects. (For interpretation of the references to colour in this figure legend, the reader is referred to the Web version of this article.)

**Fig. 6d:**
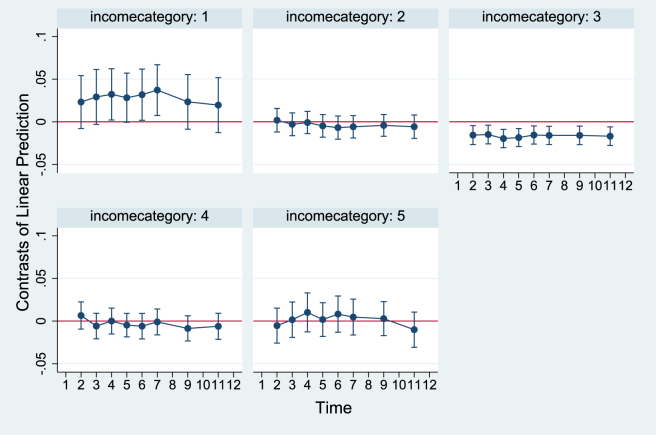
Difference in gender of average marginal effects of time on the occurrence of generalized anxiety disorders by income. Note: the horizontal red line at zero is added since we are interested in effects different from zero. Positive effects indicate that women in a certain income category have more anxiety disorders than men in the same income category, vice versa for negative effects. (For interpretation of the references to colour in this figure legend, the reader is referred to the Web version of this article.)

#### 5.4.4 Time, gender and marital status

Investigating the interaction between gender, time and marital status in Fig. 7a, Fig. 7b, Fig. 7c, Fig. 7d, we observe that married women experience significantly more depression symptoms than married men from prolonged lockdown, ceteris paribus. An explanation might be that opposed to married men, married women are generally mostly in charge of housekeeping and taking care of the children, next to any full-time or part-time job they might have, which might cause extra mental health problems when children are at home during the COVID-19 lockdown (Alon et al., 2020).

**Fig. 7a:**
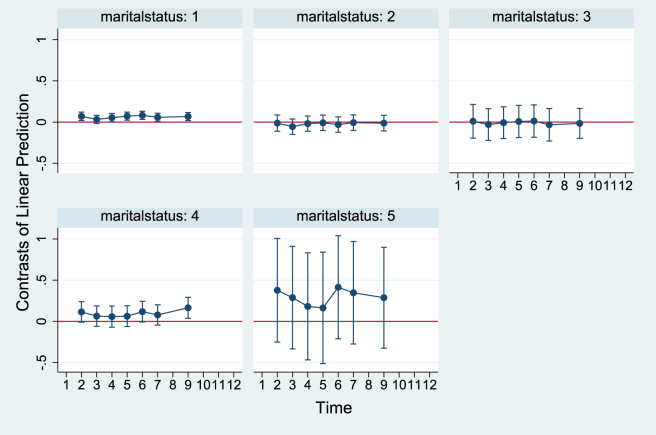
Difference in gender of average marginal effects of time on the number of depression symptoms by marital status. Note: the horizontal red line at zero is added since we are interested in effects different from zero. Positive effects indicate that women with a certain marital status have more depression symptoms than men with the same marital status, vice versa for negative effects. (For interpretation of the references to colour in this figure legend, the reader is referred to the Web version of this article.)

**Fig. 7b:**
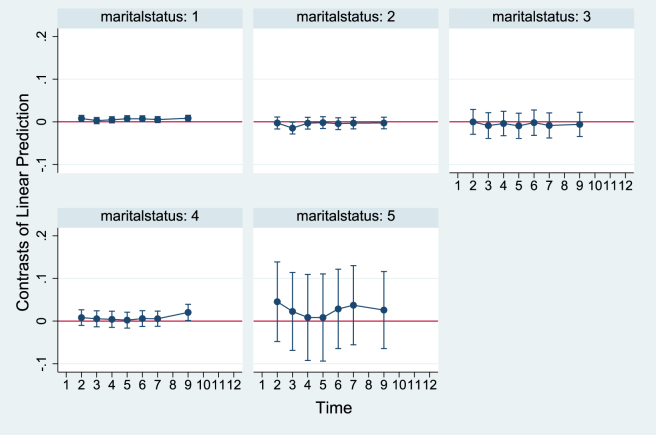
Difference in gender of average marginal effects of time on the occurrence of major depressive disorders by marital status. Note: the horizontal red line at zero is added since we are interested in effects different from zero. Positive effects indicate that women with a certain marital status have more depression disorders than men with the same marital status, vice versa for negative effects. (For interpretation of the references to colour in this figure legend, the reader is referred to the Web version of this article.)

**Fig. 7c:**
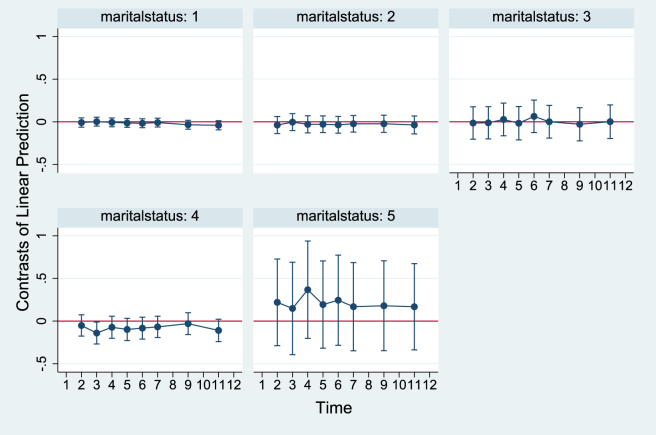
Difference in gender of average marginal effects of time on the number of anxiety symptoms by marital status. Note: the horizontal red line at zero is added since we are interested in effects different from zero. Positive effects indicate that women with a certain marital status have more anxiety symptoms than men with the same marital status, vice versa for negative effects. (For interpretation of the references to colour in this figure legend, the reader is referred to the Web version of this article.)

**Fig. 7d:**
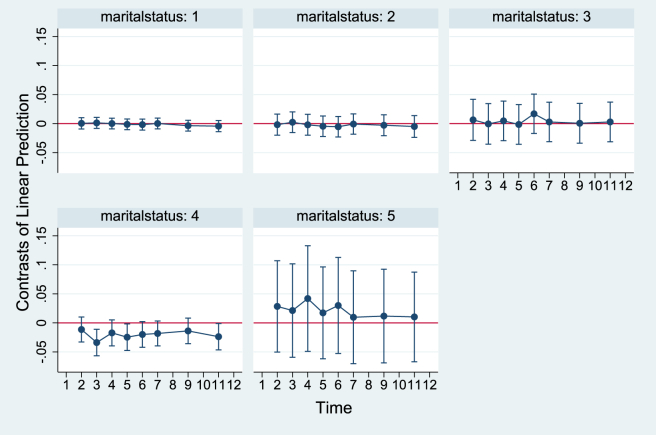
Difference in gender of average marginal effects of time on the occurrence of generalized anxiety disorders by marital status. Note: the horizontal red line at zero is added since we are interested in effects different from zero. Positive effects indicate that women with a certain marital status have more anxiety disorders than men with the same marital status, vice versa for negative effects. (For interpretation of the references to colour in this figure legend, the reader is referred to the Web version of this article.)

#### 5.4.5 Time, gender and number of children

Lastly, we inspect the interaction between gender, time and the number of children in the house, where we look at number of children aged between zero and 12, and at the number of children aged between 13 and 18. Both do not provide significant differences in mental health of gender over time, therefore, we omit these results.

## 6 Discussions and limitations

In this study we investigated the impact of the COVID-19 lockdown on gender differences in mental health using data from Northern Netherlands. During the lockdown, Northern Netherlands had very few reported COVID-19 cases, therefore, our analysis is able to highlight the impact of the lockdown itself, i.e. without the presence of COVID-19. It is worth noticing that the lockdown in the Netherlands is different from lockdown measures in other countries, since only those in contact with COVID-19 had to be quarantined. For example, the lockdown in Italy forced everyone to stay home with exceptions for necessity, work and health circumstances. When investigating mental health, we found that our sample shows a much lower percentage indicating to have a major depressive disorder or a generalized anxiety disorder during the COVID-19 lockdown than other countries, such as Italy, we reviewed in Section 2. Even though this difference might, partly, be due to differences in forms of screening of depression and anxiety since various measures are available, the difference is quite substantial. Further research might be necessary to identify exactly where these differences stem from and how these affect the results.

Furthermore, when comparing respondents and non-respondents of the COVID-19 questionnaires in Table 2, we see that respondents have significantly less depression and anxiety symptoms and disorders than non-respondents and that respondents rate their health better. As mentioned in Section 3, this indicates that we face a selection problem in the form of a healthy user bias, meaning that the respondents were healthier than non-respondents to begin with. This implies that, when not correcting for this, conclusions drawn using this sample are incorrect for the population is a whole. We checked if sample selection is still present when controlling for other characteristics. Furthermore, we also checked for attrition bias, which occurs when subjects leave during the study in a non-random way. Details on these tests can be found in Appendix 1. Fortunately, sample selection bias vanishes when controlling for other characteristics, and attrition bias is not present.

Important to notice is the fact that we used linear fixed effects models, whereas, in fact, the number of depression symptoms and the number of anxiety symptoms are count variables, and the occurrence of major depressive disorders and the occurrence of generalized anxiety are binary variables. In the last case, using a linear model might not be trustworthy since the percentage indicating to have a disorder is low. For the number of depression symptoms and the number of anxiety symptoms we use a Poisson fixed effects (a count model) to confirm the results, and for the occurrence of major depressive disorders and generalized anxiety disorders we use a Probit (Mundlak) model (a binary model) to confirm our findings. The methodology and results of these additional estimations are available upon request.

On top of this, we also defined and estimated additional models. As already mentioned in a footnote, we performed a cross-sectional analysis which confirmed the existing literature. Furthermore, to confirm the results we found, we defined and estimated a model introducing other relevant variables (such as smoke-situation and worksituation). The methodology and results of these estimations are also available upon request.

As mentioned in Section 3.1, both the mean number of number of depression symptoms and anxiety symptoms and disorders decrease when the lockdown starts and never return to their initial high state before the lockdown, which is between three and six years before the COVID-19 questionnaires. In these years a lot might have happened explaining the sudden decrease in depression and anxiety symptoms and disorders as seen in Fig. 1a and Fig. 1b. Nonetheless, for validation purposes of the results and conclusions it is important to define and estimate a model without the influence of the pre-lockdown period. Therefore, we removed this initial period and estimated the models without this period, i.e. using only data from during the COVID-19 pandemic. Performing a joint significance test for the interaction terms between gender and the time periods, as we did in the model including the pre-lockdown time period, we obtain similar results as before. This confirms the results that the lockdown causes additional mental health differences, when looking at depression and anxiety, between men and women. The methodology and results of this estimation method excluding the pre-lockdown time period are available upon request.

To sum up the models: next to the linear fixed effects model discussed here, we also defined and estimated a Poisson count model (to acknowlegde the count structure of the number of symptoms of depression and anxiety), a Probit model (to acknowlegde the binary structure of the occurrence of major depressive and generalized anxiety disorders), a cross-sectional model (to confirm existing literature with the current dataset), an extended model (to confirm the found results when introducing other relevant variables) and a model excluding the pre-COVID-19 time period (to confirm the found results using only data during the COVID-19 pandemic). The results of these other models all confirm the results found with the linear fixed effects model.

## 7 Conclusion

We investigated gender differences in the mental health impact of the COVID-19 lockdown. Women react significantly worse to the lockdown in terms of depression symptoms and disorders, but significantly better in terms of anxiety symptoms and disorders than men. Hence, the effects of the COVID-19 lockdown on mental health are worse for women regarding depression, but worse for men regarding anxiety. The fact that women experience more depression symptoms and disorders due to the lockdown, contributes to widening the existing structural gender gap (World Economic Forum, 2020). A way to diminish the mental health gender gap is for policymakers to consider providing child day care during the lockdown. Generally, women take care of the children besides any full-time or part-time job they might have, which might cause additional mental health problems when in lockdown since women need to work from home whereas their children are also at home. Hence, providing child day care during the COVID-19 lockdown is likely to reduce lockdown induced gender differences in mental health.

From the three-way interactions we found that young men experience significantly more anxiety disorders than young women stemming from prolonged lockdown. Furthermore, married women, highly educated women and women with low income experience more depression problems from prolonged lockdown than married men, highly educated men and men with low income, respectively. An example to support low-income women who face mental health issues is for policymakers to consider providing free accessible treatment or medication for depression and anxiety, such that low-income women can increase their engagement in treatment to reduce depression and anxiety symptoms and disorders, which was also suggested by Levy and O'Hara (2010). Specifically related to the COVID-19 lockdown, a way to diminish mental health related issues is to proactively offer e-mental health modules, accessible to everyone, to help people cope better with their mental health issues while staying at home.

Considering that the COVID-19 pandemic is likely to continue for some time, the results can be used to better understand how men and women react to prolonged lockdown in terms of mental health, and these findings can be used to support decision making, i.e. the findings can be taken into account when issuing new restrictions and/or lockdown measures.

## Funding

ZonMW Corona Fast-Track Grant (440.20.002.).

## Ethical statement

This study is based on data from the Lifelines Corona Research study, which was approved by the medical ethical committee of the University Medical Centre Groningen.

## Declaration of competing interest

None of the authors report a conflict of interest.

## Footnotes

1 It is worth noting that similar graphs were made using a balanced panel, i.e. with only those who answered each questionnaire, which resulted in similar patterns. Furthermore, for each different depression and anxiety symptom we also made similar graphs to observe if obvious differences between symptoms were present, which were not.

2 The same graphical analysis is performed for the occurrence of major depressive disorders and generalized anxiety disorders, which can be found in Fig. 2c and Fig. 2d. These figures are very similar to Fig. 2a and Fig. 2b, respectively.

3 In order to confirm our findings, we also estimate several other models, e.g. a Poisson count model and a probit model. We will get back to this in Section 6.

4 It is worth noticing that a cross-sectional analysis (available upon request) confirms the literature review, i.e. women, young people and individuals with low SES, experience more depression and anxiety symptoms and disorders than men, older people, and individuals with higher SES.

Appendix A Supplementary data to this article can be found online at https://doi.org/10.1016/j.ssmph.2021.100878.

## References

[bib1] Ahmad A., Rahman I., Agarwal M. (2020). Early psychosocial predictors of mental health among Indians during coronavirus disease 2019 outbreak. Journal of Health Science.

[bib2] Ahmed M.Z., Ahmed O., Aibao Z., Hanbin S., Siyu L., Ahmad A. (2020). Epidemic of COVID-19 in China and associated psychological problems. Asian Journal of Psychiatry.

[bib3] Alon T., Doepke M., Olmstead-Rumsey J., Tertilt M. (2020).

[bib4] Angelini V., Howdon D.D.H., Mierau J.O. (2018). Childhood socioeconomic status and late-adulthood mental health: Results from the survey on health, ageing and retirement in europe. Journal of Gerontology: Series B.

[bib5] Barzilay R., Moore T.M., Greenberg D.M., DiDomenico G.E., Brown L.A., White L.K., Gur R.C., Gur R.E. (2020). Resilience, covid-19-related stress, anxiety and depression during the pandemic in a large population enriched for healthcare providers. Translational Psychiatry.

[bib6] Batz-Barbarich C., Tay L., Kuykendall L., Cheung H.K. (2018). A meta-analysis of gender differences in subjective well-being: Estimating effect sizes and associations with gender inequality. Psychological Science.

[bib7] Brooks S.K., Webster R.K., Smith L.E., Woodland L., Wessely S., Greenberg N., Rubin G.J. (2020). The psychological impact of quarantine and how to reduce it: Rapid review of the evidence. The Lancet.

[bib8] Burström B., Tao W. (2020). Social determinants of health and inequalities in COVID-19. The European Journal of Public Health.

[bib9] Bussemakers C., Van Oosterhout K., Kraaykamp G., Spierings N. (2017). Women's worldwide education–employment connection: A multilevel analysis of the moderating impact of economic, political, and cultural contexts. World Development.

[bib10] Callander E.J. (2016). Pathways between health, education and income in adolescence and adulthood. Archives of Disease in Chilhood.

[bib11] Cellini N., Canale N., Mioni G., Costa S. (2020). Changes in sleep pattern, sense of time and digital media use during COVID-19 lockdown in Italy. Journal of Sleep Research.

[bib12] Daly M., Robinson E. (2021). Psychological distress and adaptation to the covid-19 crisis in the United States. Journal of Psychiatric Research.

[bib13] De Graaf R., Ten Have M., Van Gool C., Van Dorsselaer S. (2012). Prevalence of mental disorders and trends from 1996 to 2009. Results from The Netherlands mental health survey and incidence study-2. Social Psychiatry and Psychiatric Epidemiology.

[bib14] Elmer T., Mepham K., Stadtfeld C. (2020). Students under lockdown: Comparisons of students' social networks and mental health before and during the COVID-19 crisis in Switzerland. PloS One.

[bib15] Gao W., Ping S., Liu X. (2020). Gender differences in depression, anxiety, and stress among college students: A longitudinal study from China. Journal of Affective Disorders.

[bib16] Gebhard C., Regitz-Zagrosek V., Neuhauser H.K., Morgan R., Klein S.L. (2020). Impact of sex and gender on COVID-19 outcome in Europe. Biology of Sex Differences.

[bib17] González-Sanguino C., Ausín B., Castellanos M.Á., Saiz J., López-Gómez A., Ugidos C., Muñoz M. (2020). Mental health consequences during the initial stage of the 2020 Coronavirus pandemic (COVID-19) in Spain. Brain, Behavior, and Immunity.

[bib18] Gualano M.R., Moro G.L., Voglino G., Bert F., Siliquini R. (2020). Effects of covid-19 lockdown on mental health and sleep disturbances in Italy. International Journal of Environmental Research and Public Health.

[bib19] Holman E.A., Thompson R.R., Garfin D.R., Silver R.C. (2020). The unfolding covid-19 pandemic: A probability-based, nationally representative study of mental health in the United States. Science advances.

[bib20] Holmes E.A., O'Connor R.C., Perry V.H., Tracey I., Wessely S., Arseneault L., Ballard C., Christensen H., Silver R.C., Everall I., Ford T., John A., Kabir T., King K., Madan I., Michie S., Przybylski A.K., Shafran R., Sweeney A., andBullmor M. (2020). Multidisciplinary research priorities for the COVID-19 pandemic: A call for action for mental health science. Lancet Psychiatry.

[bib21] Huang Y., Zhao N. (2020). Generalized anxiety disorder, depressive symptoms and sleep quality during COVID-19 outbreak in China: A web-based cross-sectional survey. Psychiatry Research.

[bib22] Ji Kang S., In Jung S. (2020). Age-related morbidity and mortality among patients with COVID-19. Journal of Infection and Chemotherapy.

[bib23] Kapteyn A., Alessie R., Lusardi A. (2005). Explaining the wealth holdings of different cohorts: Productivity growth and Social Security. European Economic Review.

[bib24] Kernberg, American Psychiatric Association (2013).

[bib25] Khazanchi R., Beiter E.R., Gondi S., Beckman A.L., Bilinski A., Ganguli I. (2020). County-level association of social vulnerability with COVID-19 cases and deaths in the USA. Journal of General Internal Medicine.

[bib26] Kiely K.M., Brady B., Byles J. (2019). Gender, mental health and ageing. Maturitas.

[bib27] Lei L., Huang X., Zhang S., Yang J., Yang L., Xu M. (2020). Comparison of prevalence and associated factors of anxiety and depression among people affected by versus people unaffected by quarantine during the COVID-19 epidemic in Southwestern China. Medical Science Monitor.

[bib28] Levy L.B., O'Hara M.W. (2010). Psychotherapeutic interventions for depressed, low-income women: A review of the literature. Clinical Psychology Review.

[bib29] Liu C.H., Zhang E., Wong G.T.F., Hyun S., Hahm H.C. (2020). Factors associated with depression, anxiety, and ptsd symptomatology during the covid-19 pandemic: Clinical implications for us young adult mental health. Psychiatry Research.

[bib30] Mazza C., Ricci E., Biondi S., Colasanti M., Ferracuti S., Napoli C., Roma P. (2020). A Nationwide survey of psychological distress among Italian people during the COVID-19 pandemic: Immediate psychological responses and associated factors. International Journal of Environmental Research and Public Health.

[bib31] McGinn K.L., Oh E. (2017). Gender, social class, and women's employment. Current opinion in psychology.

[bib32] McIntyre K., Lanting P., Deelen P., Wiersma H., Vonk J.M., Ori A., Jankipersadsing S.A., Warmerdam R., Van Blokland I., Boulogne F., Dijkema M., Herkert J.C., Claringbould A., Bakker O., Lopera Maya E.A., Bultmann U., Zhernakova A., Reijneveld S.A., Zijlstra E., Franke L. (2021). [Forthcoming]. The Lifelines COVID-19 cohort: A questionnaire-based study to investigate COVID-19 infection and its health and societal impacts in a Dutch population-based cohort. BMJ Open.

[bib33] Meyer J., McDowell C., Lansing J., Brower C., Smith L., Tully M., Herring M. (2020). Changes in physical activity and sedentary behavior in response to covid-19 and their associations with mental health in 3052 us adults. International Journal of Environmental Research and Public Health.

[bib34] Moghanibashi-Mansourieh A. (2020). Assessing the anxiety level of Iranian general population during COVID-19 outbreak. Asian Journal of Psychiatry.

[bib35] Moreira P.S., Ferreira S., Couto B., Machado-Sousa M., Fernandez M., Raposo-Lima C., Sousa N., Pico-Perez M., Morgado P. (2020).

[bib36] Petrongolo B. (2019). The gender gap in employment and wages. Nature Human Behaviour.

[bib37] Pieh C., Budimir S., Probst T. (2020). The effect of age, gender, income, work, and physical activity on mental health during coronavirus disease (COVID-19) lockdown in Austria. Journal of Psychosomatic Research.

[bib38] Pouso S., Borja A., Fleming L.E., Gómez-Baggethun E., White M.P., Uyarra M.C. (2021). Contact with blue-green spaces during the COVID-19 pandemic lockdown beneficial for mental health. The Science of the Total Environment.

[bib39] Ritchie H., Roser M. (2018). https://ourworldindata.org/mental-health.

[bib40] Rossi R., Socci V., Talevi D., Mensi S., Niolu C., Pacitti F., Di Marco A., Rossi A., Siracusano A., Di Lorenzo G. (2020). COVID-19 pandemic and lockdown measures impacton mental health among the general population in Italy. An N= 18147 web-based survey. Frontiers in Psychiatry.

[bib41] Rudenstine S., McNeal K., Schulder T., Ettman C.K., Hernandez M., Gvozdieva K., Galea S. (2021). Depression and anxiety during the covid-19 pandemic in an urban, low-income public university sample. Journal of Traumatic Stress.

[bib42] Samarakoon S., Parinduri R.A. (2015). Does education empower women? Evidence from Indonesia. World Development.

[bib43] Silbersdorff A., Schneider K.S. (2019). Distributional regression techniques in socioeconomic research on the inequality of health with an application on the relationship between mental health and income. International Journal of Environmental Research and Public Health.

[bib44] Ueda M., Stickley A., Sueki H., Matsubayashi T. (2020).

[bib45] UNESCO Institute for Statistics (2012).

[bib46] Wang C., Pan R., Wan X., Tan Y., Xu L., Ho C.S., Ho R.C. (2020). Immediate psychological responses and associated factors during the initial stage of the 2019 coronavirus disease (COVID-19) epidemic among the general population in China. International Journal of Environmental Research and Public Health.

[bib47] Wang X., Hegde S., Son C., Keller B., Smith A., Sasangohar F. (2020). Investigating mental health of US college students during the COVID-19 pandemic: Cross-sectional survey study. Journal of Medical Internet Research.

[bib48] White R.G., Van der Boor C. (2020). Impact of the COVID-19 pandemic and initial period of lockdown on the mental health and well-being of adults in the UK. BJPsych Open.

[bib49] WHO (2020).

[bib50] World Economic Forum (2020).

[bib51] Zhang S.X., Wang Y., Afshar Jahanshahi A., Jia J., Haensel Schmitt V.G. (2020).

