# Supplementary material for: Gender differences in the mental health impact of the COVID-19 lockdown: Longitudinal evidence from the Netherlands
Source: SSM Popul Health. 2021 Jul 24;15:100878. doi: 10.1016/j.ssmph.2021.100878 (PMC8387764; doi:10.1016/j.ssmph.2021.100878)
Supplement: Multimedia component 1 [file mmc1.pdf]

Appendices

1 Selection bias

1.1 Sample selection bias

From the sample characteristics we noted that the number of depression and anxiety symptoms, and the percentage of the sample having major depressive and generalized anxiety disorders are significantly different for respondents and non-respondents of the COVID-19 questionnaires. This is measured at time point zero, i.e. before the COVID-19 pandemic. We also observed that respondents were significantly more often ex-smokers and significantly less often current smokers, and that respondents have a significantly higher SES than non-respondents, as can be seen by highest education achieved and net income per month. Hence, we face a sample selection problem in the form of a healthy user bias, meaning that the respondents were healthier than non-respondents to begin with, i.e. before the lockdown even started. If sample selection bias is present, we do not have a random sample, meaning that the sample is not representative for the population and conclusions drawn from this sample are incorrect for the whole population. Furthermore, sample selection may also affect the estimation of relationships between variables which results in inaccurate estimates. A result of sample selection bias might be that we wrongly conclude for the population that mental health is not as bad as we expect during the COVID-19 pandemic, whereas, in fact, this conclusion is based on a sample with healthy subjects reporting less mental health problems than the overall population of which the sample is drawn.

We need to examine if this selection problem is still present when controlling for other characteristics. We perform a regression on these variables before the lockdown using the characteristics mentioned in the table in the Data section as independent variables (except, of course, the variables of depression and anxiety, which we use as dependent variables). We include a variable indicating whether the individual responded to at least one of the COVID-19 questionnaires or not (*Respondent*). For each of the above mentioned dependent variables, we find insignificant coefficients for the variable *Respondent* on a 5% significance level, meaning that we cannot reject the null hypothesis that the coefficient for *Respondent* is equal zero, indicating that the selection problem vanishes when controlling for other characteristics. The results of variable *Respondent* can be found in Table 1 where we excluded all other independent variables for convenience.

Table 1: Sample selection bias test.

	(1) Depr. symptoms	(2) Depr. disorder	(3) Anx. symptoms	(4) Anx. disorder
Respondent	0.0624	0.00834	0.159	0.0233
	(0.101)	(0.0144)	(0.113)	(0.0200)
N	27177	27177	27177	27177

Standard errors in parentheses

* $p < 0.05$, ** $p < 0.01$, *** $p < 0.001$

1.2 Attrition bias

Other than sample selection bias, we might suffer from a different selection bias, namely attrition bias. Whereas sample selection bias occurs before the study starts, attrition bias occurs when we have loss of participants during the study, i.e. individuals stop filling in COVID-19 questionnaires. Even if no selection bias is present at the first questionnaire, after some questionnaires the respondents may differ significantly from subjects no longer responding. Attrition bias is present when differences exist between individuals leaving the study at some point and individuals who continue the study. In our case, attrition bias is present when, for example, individuals are too depressed to fill in the COVID-19 questionnaires on how depressed they are. Consequences are similar to the consequences of sample selection bias: The sample is no longer representative for the population and, hence, conclusions drawn from the sample cannot be applied to this population, i.e. the results are biased.

In line with Kapteyn et al. (2005), we examine if we have a problem of attrition bias by constructing dummy variables for each subject, equal to one if the individual filled out the COVID-19 questionnaire in this time period and the individual filled out a COVID-19 questionnaire in a next period; and the dummy variable is equal to zero if the individual filled out the COVID-19 questionnaire in this time period, but then drops out of the sample. We include these dummy variables in Model 1 as described in Section 4. Next, we use an F-test to test joint significance of these variables. If these variables are jointly significant, we have evidence of attrition bias. Performing F-tests we obtain the P-values as given in Table 2. Fortunately, we do not suffer from attrition bias.

Table 2: Results of F-tests for attrition bias.

Dependent variable	P-values	Attrition bias (5% level)
Number of depression symptoms	0.5758	No
Number of anxiety symptoms	0.4037	No
Major depressive disorder	0.7798	No
Generalized anxiety disorder	0.3973	No

2 Results base model

Table 3: Results base model.

	(1)<br>Depr. symptoms	(2)<br>Depr. disorder	(3)<br>Anx. symptoms	(4)<br>Anx. disorder
Time 2	-0.0707	0.00722	-0.0757	-0.0246
	(0.120)	(0.0189)	(0.119)	(0.0217)
Time 3	-0.0229	0.0188	-0.106	-0.0170
	(0.117)	(0.0192)	(0.121)	(0.0217)
Time 4	-0.137	-0.00257	-0.0161	-0.00267
	(0.109)	(0.0165)	(0.117)	(0.0210)
Time 5	-0.143	-0.00243	-0.0547	-0.00823
	(0.110)	(0.0174)	(0.111)	(0.0204)
Time 6	-0.121	0.00243	0.0969	0.0215
	(0.109)	(0.0177)	(0.124)	(0.0235)
Time 7	-0.109	0.00244	-0.164	-0.0305
	(0.121)	(0.0188)	(0.113)	(0.0207)
Time 8	-0.0797	0.0217	-0.164	-0.0298
	(0.114)	(0.0192)	(0.115)	(0.0212)
Time 9	-0.0975	0.0221	-0.0538	-0.00939
	(0.130)	(0.0199)	(0.120)	(0.0217)
Time 10	-0.0726	0.00850	-0.0221	0.00168
	(0.119)	(0.0182)	(0.117)	(0.0213)
Time 11	-0.113	0.0111	-0.119	-0.0265
	(0.122)	(0.0167)	(0.123)	(0.0225)
Time 12	-0.109	0.0262	-0.141	-0.0301
	(0.124)	(0.0188)	(0.115)	(0.0210)
**Woman** ×				

Table 3 continued: Results base model.

Time 2	0.0439*	0.00395	-0.0317	-0.00528
	(0.0179)	(0.00257)	(0.0186)	(0.00333)
Time 3	0.00272	-0.00177	-0.0423*	-0.00819*
	(0.0172)	(0.00255)	(0.0181)	(0.00327)
Time 4	0.0233	0.000568	-0.0450*	-0.00820*
	(0.0171)	(0.00249)	(0.0180)	(0.00320)
Time 5	0.0355*	0.00168	-0.0465**	-0.00909**
	(0.0168)	(0.00244)	(0.0174)	(0.00312)
Time 6	0.0491**	0.00288	-0.0399*	-0.00751*
	(0.0167)	(0.00248)	(0.0180)	(0.00322)
Time 7	0.0329	0.00179	-0.0410*	-0.00753*
	(0.0170)	(0.00249)	(0.0179)	(0.00317)
Time 8	0.0381*	0.00403	-0.0320	-0.00584
	(0.0170)	(0.00246)	(0.0179)	(0.00319)
Time 9	0.0463**	0.00485	-0.0492**	-0.00896**
	(0.0170)	(0.00252)	(0.0180)	(0.00318)
Time 10	0.0165	-0.000141	-0.0572**	-0.00961**
	(0.0173)	(0.00256)	(0.0189)	(0.00336)
Time 11	0.0118	-0.0000953	-0.0665***	-0.0113***
	(0.0179)	(0.00264)	(0.0185)	(0.00328)
Time 12	0.0199	-0.000607	-0.0533**	-0.00912**
	(0.0175)	(0.00258)	(0.0184)	(0.00325)
**Age 31-50** ×				
Time 2	-0.0434	-0.0188	-0.00863	0.00103
	(0.0766)	(0.0124)	(0.0881)	(0.0147)
Time 3	-0.0504	-0.0212	-0.00985	-0.00104
	(0.0756)	(0.0116)	(0.0893)	(0.0152)
Time 4	-0.0472	-0.0149	-0.0536	-0.0120
	(0.0761)	(0.0105)	(0.0841)	(0.0143)
Time 5	-0.0727	-0.0223	-0.0705	-0.0124
	(0.0822)	(0.0123)	(0.0903)	(0.0148)
Time 6	-0.0500	-0.0196	-0.100	-0.0188
	(0.0793)	(0.0118)	(0.0916)	(0.0161)
Time 7	-0.126	-0.0235	0.0358	0.00800

Table 3 continued: Results base model.

	(0.0837)	(0.0128)	(0.0862)	(0.0148)
Time 8	-0.131	-0.0346*	0.0515	0.00668
	(0.0809)	(0.0136)	(0.0851)	(0.0147)
Time 9	-0.0902	-0.0246	-0.0339	-0.00411
	(0.0896)	(0.0132)	(0.0865)	(0.0148)
Time 10	-0.0941	-0.0205	-0.0800	-0.0206
	(0.0806)	(0.0120)	(0.0854)	(0.0149)
Time 11	-0.0951	-0.0163	-0.00146	-0.00158
	(0.0839)	(0.0113)	(0.0859)	(0.0150)
Time 12	-0.0117	-0.0204	0.0590	0.0136
	(0.0803)	(0.0117)	(0.0829)	(0.0141)
**Age 51-65** ×				
Time 2	-0.0454	-0.0156	-0.0272	-0.00237
	(0.0753)	(0.0123)	(0.0869)	(0.0144)
Time 3	-0.0403	-0.0174	-0.0337	-0.00511
	(0.0744)	(0.0114)	(0.0882)	(0.0150)
Time 4	-0.0690	-0.0142	-0.0518	-0.0114
	(0.0750)	(0.0103)	(0.0829)	(0.0141)
Time 5	-0.0652	-0.0192	-0.0917	-0.0166
	(0.0813)	(0.0122)	(0.0893)	(0.0146)
Time 6	-0.0633	-0.0169	-0.124	-0.0242
	(0.0781)	(0.0116)	(0.0905)	(0.0159)
Time 7	-0.124	-0.0216	0.0131	0.00297
	(0.0828)	(0.0127)	(0.0850)	(0.0146)
Time 8	-0.104	-0.0280*	0.0368	0.00277
	(0.0801)	(0.0135)	(0.0840)	(0.0145)
Time 9	-0.0766	-0.0191	-0.0358	-0.00605
	(0.0889)	(0.0131)	(0.0855)	(0.0147)
Time 10	-0.0774	-0.0167	-0.0841	-0.0223
	(0.0796)	(0.0118)	(0.0842)	(0.0147)
Time 11	-0.0703	-0.0101	-0.0206	-0.00438
	(0.0830)	(0.0111)	(0.0847)	(0.0148)
Time 12	-0.0182	-0.0186	0.0315	0.00861
	(0.0793)	(0.0116)	(0.0816)	(0.0139)

Table 3 continued: Results base model.

**Age 66 and older ×**				
Time 2	-0.0421	-0.0139	0.0519	0.0118
	(0.0758)	(0.0123)	(0.0867)	(0.0144)
Time 3	-0.0333	-0.0184	0.0542	0.00904
	(0.0747)	(0.0114)	(0.0881)	(0.0150)
Time 4	-0.0293	-0.0111	0.0290	0.00243
	(0.0754)	(0.0103)	(0.0829)	(0.0141)
Time 5	-0.0329	-0.0156	0.00339	-0.000266
	(0.0817)	(0.0122)	(0.0893)	(0.0146)
Time 6	-0.00523	-0.0111	-0.0275	-0.00825
	(0.0786)	(0.0116)	(0.0907)	(0.0160)
Time 7	-0.0961	-0.0190	0.110	0.0199
	(0.0832)	(0.0127)	(0.0851)	(0.0146)
Time 8	-0.0716	-0.0269*	0.134	0.0197
	(0.0805)	(0.0136)	(0.0841)	(0.0145)
Time 9	-0.0350	-0.0161	0.0455	0.00879
	(0.0892)	(0.0132)	(0.0855)	(0.0147)
Time 10	-0.0231	-0.0127	-0.00900	-0.00955
	(0.0800)	(0.0118)	(0.0842)	(0.0147)
Time 11	-0.0310	-0.00803	0.0717	0.0114
	(0.0833)	(0.0111)	(0.0849)	(0.0148)
Time 12	0.00900	-0.0164	0.132	0.0261
	(0.0796)	(0.0116)	(0.0817)	(0.0139)
**Lower secondary vocational or junior general secondary education ×**				
Time 2	-0.00455	-0.00544	-0.0764	-0.00217
	(0.0933)	(0.0144)	(0.0797)	(0.0160)
Time 3	-0.0600	-0.0120	-0.0461	-0.00516
	(0.0899)	(0.0154)	(0.0796)	(0.0152)
Time 4	0.0601	0.00438	-0.104	-0.0133
	(0.0783)	(0.0130)	(0.0803)	(0.0154)
Time 5	0.0581	0.00654	-0.0274	-0.00219

Table 3 continued: Results base model.

	(0.0732)	(0.0122)	(0.0647)	(0.0142)
Time 6	0.0261	-0.00105	-0.121	-0.0204
	(0.0758)	(0.0135)	(0.0827)	(0.0170)
Time 7	0.0877	0.00439	-0.0363	-0.00350
	(0.0884)	(0.0139)	(0.0725)	(0.0146)
Time 8	0.0412	-0.00712	-0.0444	0.0000490
	(0.0798)	(0.0134)	(0.0767)	(0.0152)
Time 9	0.00172	-0.0210	-0.0750	-0.0109
	(0.0942)	(0.0149)	(0.0820)	(0.0158)
Time 10	-0.0156	-0.00646	-0.0354	-0.00175
	(0.0880)	(0.0140)	(0.0790)	(0.0153)
Time 11	0.0481	-0.00889	-0.00529	0.00617
	(0.0885)	(0.0125)	(0.0885)	(0.0168)
Time 12	-0.0149	-0.0179	-0.0524	-0.00478
	(0.0952)	(0.0149)	(0.0785)	(0.0154)
**Secondary vocational or senior general secondary education ×**				
Time 2	0.0302	-0.00272	-0.108	-0.00746
	(0.0933)	(0.0144)	(0.0799)	(0.0160)
Time 3	-0.0310	-0.0102	-0.0724	-0.0112
	(0.0899)	(0.0153)	(0.0796)	(0.0152)
Time 4	0.114	0.0101	-0.134	-0.0190
	(0.0783)	(0.0129)	(0.0806)	(0.0154)
Time 5	0.110	0.0117	-0.0481	-0.00704
	(0.0729)	(0.0122)	(0.0648)	(0.0143)
Time 6	0.0800	0.00386	-0.153	-0.0273
	(0.0756)	(0.0134)	(0.0828)	(0.0170)
Time 7	0.131	0.0103	-0.0540	-0.00769
	(0.0882)	(0.0139)	(0.0724)	(0.0146)
Time 8	0.0929	-0.00109	-0.0598	-0.00451
	(0.0796)	(0.0133)	(0.0769)	(0.0152)
Time 9	0.0629	-0.0143	-0.103	-0.0163

Table 3 continued: Results base model.

	(0.0942)	(0.0149)	(0.0821)	(0.0158)
Time 10	0.0561	0.00184	-0.0659	-0.00784
	(0.0878)	(0.0139)	(0.0789)	(0.0153)
Time 11	0.106	-0.00302	-0.0494	-0.00214
	(0.0882)	(0.0124)	(0.0886)	(0.0169)
Time 12	0.0417	-0.0125	-0.0642	-0.00728
	(0.0951)	(0.0149)	(0.0785)	(0.0154)
**Higher vocational or university education** ×				
Time 2	0.0476	0.000994	-0.0619	0.00180
	(0.0936)	(0.0145)	(0.0800)	(0.0161)
Time 3	0.00533	-0.00417	-0.0414	-0.00332
	(0.0901)	(0.0154)	(0.0796)	(0.0152)
Time 4	0.143	0.0142	-0.0984	-0.0114
	(0.0786)	(0.0130)	(0.0806)	(0.0155)
Time 5	0.129	0.0147	-0.0113	-0.000433
	(0.0732)	(0.0122)	(0.0649)	(0.0143)
Time 6	0.114	0.00874	-0.106	-0.0188
	(0.0759)	(0.0135)	(0.0828)	(0.0170)
Time 7	0.160	0.0129	-0.0396	-0.00287
	(0.0886)	(0.0139)	(0.0725)	(0.0146)
Time 8	0.127	0.00310	-0.0421	-0.000224
	(0.0799)	(0.0134)	(0.0770)	(0.0152)
Time 9	0.0732	-0.0133	-0.0698	-0.00936
	(0.0944)	(0.0149)	(0.0823)	(0.0158)
Time 10	0.0583	0.00229	-0.0453	-0.00260
	(0.0881)	(0.0140)	(0.0790)	(0.0153)
Time 11	0.129	-0.00101	-0.0241	0.00306
	(0.0885)	(0.0125)	(0.0887)	(0.0169)
Time 12	0.0517	-0.0103	-0.0479	-0.00297
	(0.0954)	(0.0149)	(0.0785)	(0.0154)

Table 3 continued: Results base model.

**Income between €1000,- and €2000,-** ×				
Time 2	0.0420	0.000825	0.0485	0.00662
	(0.0287)	(0.00420)	(0.0304)	(0.00540)
Time 3	0.0409	0.00288	0.0313	0.00277
	(0.0280)	(0.00418)	(0.0307)	(0.00547)
Time 4	0.00378	-0.00253	0.00784	0.000437
	(0.0269)	(0.00396)	(0.0299)	(0.00523)
Time 5	0.00731	0.00102	0.0242	0.00327
	(0.0272)	(0.00398)	(0.0298)	(0.00525)
Time 6	-0.00135	-0.000508	-0.0101	-0.00232
	(0.0275)	(0.00400)	(0.0305)	(0.00537)
Time 7	0.0269	0.00178	0.0147	0.00197
	(0.0280)	(0.00405)	(0.0300)	(0.00527)
Time 8	-0.00370	-0.00316	-0.00698	-0.00232
	(0.0279)	(0.00404)	(0.0306)	(0.00537)
Time 9	0.0162	0.00114	0.0167	0.000990
	(0.0274)	(0.00397)	(0.0303)	(0.00532)
Time 10	0.0102	-0.00111	0.00967	-0.000350
	(0.0277)	(0.00416)	(0.0310)	(0.00547)
Time 11	-0.00176	-0.00469	0.0156	0.00408
	(0.0288)	(0.00424)	(0.0307)	(0.00535)
Time 12	0.000791	-0.00391	-0.00743	-0.00284
	(0.0283)	(0.00421)	(0.0309)	(0.00541)
**Income between €2000,- and €3000,-** ×				
Time 2	0.0739*	0.00734	0.0846**	0.0121*
	(0.0298)	(0.00433)	(0.0317)	(0.00563)
Time 3	0.0532	0.00483	0.0812*	0.0106
	(0.0291)	(0.00430)	(0.0319)	(0.00571)
Time 4	0.0218	0.000502	0.0656*	0.00919
	(0.0281)	(0.00407)	(0.0309)	(0.00544)

Table 3 continued: Results base model.

Time 5	0.0371	0.00526	0.0502	0.00770
	(0.0280)	(0.00404)	(0.0306)	(0.00541)
Time 6	0.0154	0.00489	0.0258	0.00314
	(0.0284)	(0.00415)	(0.0313)	(0.00554)
Time 7	0.0301	0.00415	0.0642*	0.0104
	(0.0290)	(0.00418)	(0.0310)	(0.00546)
Time 8	0.0142	0.000963	0.0410	0.00553
	(0.0290)	(0.00416)	(0.0319)	(0.00563)
Time 9	0.0546	0.00816*	0.0621*	0.00848
	(0.0286)	(0.00409)	(0.0316)	(0.00557)
Time 10	0.0435	0.00378	0.0625	0.00825
	(0.0289)	(0.00429)	(0.0327)	(0.00578)
Time 11	0.0337	0.00200	0.0517	0.00929
	(0.0301)	(0.00443)	(0.0318)	(0.00556)
Time 12	0.0370	0.00271	0.0538	0.00727
	(0.0295)	(0.00433)	(0.0322)	(0.00566)
**Income between €3000,- and €4000,-** ×				
Time 2	0.0588	0.00496	0.104**	0.0189**
	(0.0337)	(0.00481)	(0.0353)	(0.00624)
Time 3	0.0453	0.00123	0.0873*	0.0134*
	(0.0330)	(0.00481)	(0.0350)	(0.00622)
Time 4	0.00916	-0.00261	0.0824*	0.0150*
	(0.0320)	(0.00466)	(0.0346)	(0.00606)
Time 5	0.0207	0.00321	0.0533	0.00992
	(0.0316)	(0.00452)	(0.0333)	(0.00585)
Time 6	-0.0000156	0.00136	0.0457	0.0102
	(0.0319)	(0.00467)	(0.0343)	(0.00609)
Time 7	0.0387	0.00378	0.0934**	0.0173**
	(0.0329)	(0.00473)	(0.0347)	(0.00609)
Time 8	0.0180	0.000547	0.0571	0.0106
	(0.0329)	(0.00469)	(0.0350)	(0.00614)
Time 9	0.0457	0.00619	0.0732*	0.0123*

Table 3 continued: Results base model.

	(0.0322)	(0.00468)	(0.0349)	(0.00607)
Time 10	0.0466	0.00322	0.0669	0.0122
	(0.0335)	(0.00490)	(0.0360)	(0.00634)
Time 11	0.0100	-0.00133	0.0602	0.0126*
	(0.0347)	(0.00507)	(0.0359)	(0.00618)
Time 12	0.0338	0.000614	0.0551	0.00806
	(0.0332)	(0.00485)	(0.0358)	(0.00617)
**Income more than €4000,- ×**				
Time 2	0.0507	0.00524	0.116**	0.0162*
	(0.0387)	(0.00541)	(0.0390)	(0.00692)
Time 3	0.0468	0.00505	0.122**	0.0171*
	(0.0384)	(0.00563)	(0.0390)	(0.00695)
Time 4	0.0382	0.00293	0.149***	0.0244***
	(0.0376)	(0.00547)	(0.0401)	(0.00710)
Time 5	0.0367	0.00446	0.0938*	0.0149*
	(0.0376)	(0.00534)	(0.0372)	(0.00663)
Time 6	0.0229	0.00279	0.0739	0.0115
	(0.0377)	(0.00538)	(0.0382)	(0.00689)
Time 7	0.0376	0.00423	0.116**	0.0188**
	(0.0379)	(0.00547)	(0.0386)	(0.00688)
Time 8	-0.00234	0.00191	0.0940*	0.0132
	(0.0372)	(0.00528)	(0.0387)	(0.00678)
Time 9	0.0498	0.00845	0.0987**	0.0149*
	(0.0379)	(0.00540)	(0.0383)	(0.00674)
Time 10	0.0407	0.00566	0.117**	0.0179*
	(0.0384)	(0.00570)	(0.0404)	(0.00727)
Time 11	0.0237	-0.000505	0.109**	0.0173*
	(0.0401)	(0.00567)	(0.0397)	(0.00695)
Time 12	0.0386	0.00242	0.0810*	0.0115
	(0.0386)	(0.00558)	(0.0388)	(0.00686)
_cons	0.211***	0.0214***	0.259***	0.0429***
	(0.00595)	(0.000858)	(0.00659)	(0.00117)

Table 3 continued: Results base model.

N	290808	290808	290730	291086

Standard errors in parentheses, clustered at individual level.

* $p < 0.05$, ** $p < 0.01$, *** $p < 0.001$

References

Kapteyn, A., R. Alessie, and A. Lusardi (2005). Explaining the wealth holdings of different cohorts: Productivity growth and Social Security. *European Economic Review 49*(4), 1361–1391.
